# AAV vector-mediated in vivo reprogramming into pluripotency

**DOI:** 10.1038/s41467-018-05059-x

**Published:** 2018-07-09

**Authors:** Elena Senís, Lluc Mosteiro, Stefan Wilkening, Ellen Wiedtke, Ali Nowrouzi, Saira Afzal, Raffaele Fronza, Henrik Landerer, Maria Abad, Dominik Niopek, Manfred Schmidt, Manuel Serrano, Dirk Grimm

**Affiliations:** 1 Virus-Host Interaction Group, Department of Infectious Diseases/Virology, Heidelberg University Hospital, Cluster of Excellence CellNetworks, Heidelberg, 69120 Germany; 2 BioQuant, University of Heidelberg, Heidelberg, 69120 Germany; 3 Cellular Plasticity and Cancer Group, Vall d’Hebron Institute of Oncology (VHIO), Barcelona, 08035 Spain; 4 Tumor Suppression Group, Spanish National Cancer Research Centre (CNIO), Madrid, 28029 Spain; 5 Department of Translational Oncology, National Center for Tumor Diseases (NCT) and German Cancer Research Center (DKFZ), Heidelberg, 69120 Germany; 6 GeneWerk GmbH, Heidelberg, 69120 Germany; 7 Institute for Pharmacy and Molecular Biotechnology (IPMB), Bioinformatics Division, University of Heidelberg, Heidelberg, 6 Germany; 8 Theoretical Bioinformatics Division, German Cancer Research Center, Heidelberg, 69120 Germany; 9 Institute for Research in Biomedicine (IRB Barcelona), Barcelona Institute of Science and Technology (BIST), Catalan Institution for Research and Advanced Studies (ICREA), Barcelona, 08028 Spain; 10 German Center for Infection Research (DZIF), Partner site Heidelberg, Heidelberg, 69120 Germany

## Abstract

In vivo reprogramming of somatic cells into induced pluripotent stem cells (iPSC) holds vast potential for basic research and regenerative medicine. However, it remains hampered by a need for vectors to express reprogramming factors (Oct-3/4, Klf4, Sox2, c-Myc; OKSM) in selected organs. Here, we report OKSM delivery vectors based on pseudotyped Adeno-associated virus (AAV). Using the AAV-DJ capsid, we could robustly reprogram mouse embryonic fibroblasts with low vector doses. Swapping to AAV8 permitted to efficiently reprogram somatic cells in adult mice by intravenous vector delivery, evidenced by hepatic or extra-hepatic teratomas and iPSC in the blood. Notably, we accomplished full in vivo reprogramming without c-Myc. Most iPSC generated in vitro or in vivo showed transcriptionally silent, intronic or intergenic vector integration, likely reflecting the increased host genome accessibility during reprogramming. Our approach crucially advances in vivo reprogramming technology, and concurrently facilitates investigations into the mechanisms and consequences of AAV persistence.

## Abstract (web summary)

In vivo reprogramming of somatic cells is hampered by the need for vectors to express the OKSM factors in selected organs. Here the authors report new AAV-based vectors capable of in vivo reprogramming at low doses.

## Introduction

Reprogramming of cultured somatic cells into induced pluripotent stem cells (iPSC) with a cocktail of transcription factors—originally Oct-3/4 (O), Klf4 (K), Sox2 (S) and c-Myc (M)^1^—has become routine methodology, following a seminal publication a decade ago^2–4^. Since then, numerous efforts have been made to translate this key technology into living animals, fueled by two major expectations. First, one can anticipate that tools for in vivo reprogramming will help to dissect the intricate microenvironment that governs cellular (de-)differentiation in healthy or diseased multicellular organisms. Second, when stringently and spatio-temporally controlled, methods to (de-)differentiate somatic cells in vivo and in situ will enable novel, clinically relevant stratagems in regenerative medicine.

Illustrating this potential, several groups including our own^5^ have recently successfully established in vivo reprogramming technologies and harnessed these to study cellular plasticity in mice, yielding important implications for mammalian aging, tissue regeneration and cancer development. These comprise our discovery in OKSM-transgenic mice that cellular senescence or lung injury create a tissue context which favors in vivo reprogramming in neighboring cells, possibly involving the senescence-associated secretory phenotype (SASP) and secretion of interleukin-6 (IL-6)^6^. A similar paracrine, cell-non-autonomous effect including accumulation of senescent cells, enhanced cellular plasticity as well as teratoma and dysplasia formation was noted by Chiche et al.^7^ in OKSM-transgenic mice with acute or chronic muscle damage.

Moreover, we^6^ and others^7^ consistently noted a higher incidence of teratomas in older mice, implying that natural senescence during aging likewise increases cellular plasticity in vivo. Intriguingly, seminal work^8^ from the Belmonte group shows that, vice versa, cyclic induction of in vivo OKSM expression in transgenic mice can extend their lifespan and ameliorate hallmarks of aging. This process of “molecular rejuvenation” or “resetting of the aging clock” is characterized by improvements in the nuclear envelope structure of OKSM-expressing cells, reduction of DNA damage, and higher resistance of mice to metabolic disease and muscle injury^8^.

Other studies illustrate the power of in vivo reprogramming to untangle the molecular and cellular mechanisms of cancer initiation and progression to invasive phenotypes^9,10^. Ohnishi et al.^9^ reported that premature termination of inducible OKSM expression in transgenic mice resulted in kidney neoplasia resembling Wilms tumor, suggesting the use of in vivo reprogramming to model cancer development. Marion et al.^11^ found that OKSM induction in transgenic mice triggered telomere elongation as well as upregulation of telomere protein TRF1 and telomerase RNA *Terc*. Interestingly, similar telomere changes were concurrently observed in a mouse model of pancreatic ductal adenocarcinoma. Finally, Tomokuni et al.^12^ injected Sendai viral OKSM expression vectors into mouse livers and uncovered that *Kras* activation or *p53* deficiency facilitate in vivo reprogramming of liver cells, congruent with our own data^6^.

Concomitantly, the power and promise of in vivo reprogramming for regenerative medicine is showcased by encouraging proofs-of-concept, ranging from OKSM-mediated interlineage reprogramming—via plastic intermediate states—of cultured human fibroblasts into various cell types (smooth muscle^13^, endothelial^14^, mesodermal progenitor^15^, or neural progenitor^16–18^), to exciting newest data that in vivo OKSM expression can trigger cell and tissue regeneration in different organs in mice. In pioneering work, the Kostarelos group delivered OKSM-expressing plasmids to liver or skeletal muscle of adult mice, using high-pressure or intramuscular injection, respectively^19–21^. In the liver, this led to transient upregulation of pluripotency markers concurrent with a downregulation of hepatocyte markers, indicative of successful in vivo reprogramming^20^. Indeed, extracted cells showed pluripotent characteristics including teratoma formation in immunocompromised mice^19^. Similarly, local injection of OKSM-encoding plasmids into the *gastrocnemius* muscle led to induction of proliferative, pluripotent-like cells in situ and accelerated tissue regeneration in the surgically induced model of skeletal muscle injury^21^. The effects were again transient, suggesting that the reprogrammed cells had eventually differentiated and integrated into the surrounding muscle tissue.

Others intracranially injected OSKM-encoding retroviruses into mice with experimental brain injury^22^. This resulted in in vivo reprogramming of microglia and other cells to stem cell-like cells and their subsequent differentiation into functional neurons in the neurocortex. Notably, unlike in the studies that used plasmid DNA injection for OKSM expression^19–21^, mice developed teratomas in the brain long after retroviral OKSM delivery^22^, reminiscent of findings after persistent OKSM expression in transgenic mice^5,8,9^. In contrast, Seo et al. observed neither dysplasia nor tumors in OKSM-transgenic mice when in vivo reprogramming was triggered in the lateral ventricle via doxycycline infusion^23^. Instead, this led to generation of astrocytes and neuronal progenitors as well as behavioral functional restoration after ischemic brain injury.

Collectively, these reports clearly illustrate the vast potential of in vivo reprogramming technology for basic or applied research and thus created considerable enthusiasm^24–28^. At the same time, they highlight necessary improvements that will enable a broader application, namely, (i) a need for new means for in vivo OKSM delivery in a versatile, specific, potent and clinically acceptable manner, and, once established, (ii) improvements to the safety of the overall approach. As noted, to date, the majority of in vivo reprogramming studies were conducted in transgenic mice carrying drug-inducible, often polycistronic expression cassettes that permit rapid and potent induction of OKSM or of the individual factors^5–9,23,29–32^. While useful for basic iPSC research, a common caveat is their limited versatility due to the need for transgenic mouse strains. Moreover, OKSM expression is driven from promoters lacking cell specificity, causing simultaneous in vivo reprogramming in multiple organs upon promoter induction and thus complicating downstream phenotypic analyses. This was partially overcome by groups who transiently expressed OKSM in mice from plasmid DNA^20,21^, Sendai viral vectors^12^ or retroviral vectors^22^. However, broader applicability in other organs or species comprising humans is hampered by (i) a restriction of local DNA injection to a few selected organs such as the liver or the muscle, (ii) technically demanding and clinically undesirable delivery methods (hydrodynamic tail vein injection (DNA)^20^, laparotomy and infusion into liver lobes (Sendai vectors)^12^, or intracranial injection (retroviral vectors)^22^), and (iii) a limitation to actively dividing cells (retroviral vectors)^22^. Consequently, there remains a great demand for novel OKSM expression and delivery systems that overcome the current bottlenecks and, hence, facilitate a wider evaluation of in vivo reprogramming in different mammalian species, up to clinical translation in humans.

Here, we thus explored Adeno-associated viruses (AAV)^33^ as vectors for in vivo OKSM delivery that complement and expand the existing repertoire of tools, while offering a unique combination of assets including apathogenicity, independence of cell division, amenability to genetic engineering and excellent safety in humans, as documented in over 160 clinical trials^34,35^. Of note, two prior studies used conventional AAV vectors with single-stranded (ss) OKSM-encoding DNA genomes to reprogram cultured cells and achieved efficiencies of up to 0.1%, but the in vivo capacity of these vectors was never investigated^36,37^. In the present study, we demonstrate that when equipped with a combination of robust capsid, promoter, genome structure (self-complementary^38–40^ instead of single-stranded) and OKSM cDNAs, AAV vectors can mediate highly efficient and wide-spread in vivo reprogramming in adult mice, from a single peripheral low-pressure administration. Our findings should motivate numerous applications of AAV vector-mediated in vivo reprogramming and should foster consecutive attempts to maximize the safety of this technology, thus ideally accelerating the clinical translation of this exciting concept into humans.

## Results

### Design and validation of self-complementary AAV-OKSM vectors

Traditional AAV vectors contain a ssDNA genome of up to 5 kb in length which, in order to mediate transgene expression in the target cell, has to undergo conversion to a double-stranded (ds)DNA molecule. As this process is relatively slow and inefficient, we and others developed and now preferentially use an alternative vector configuration, the so-called “self-complementary” (sc)AAV vector genomes^38–42^. These carry a mutation in one of the two viral DNA packaging and replication signals, resulting in an encapsidated vector genome comprising two inverted copies of the same transgene (see Supplementary Fig. 1 for details). In the transduced cell, the latter rapidly anneal to give rise to an expression-competent dsDNA, thus overcoming the slow kinetics and limited efficiency of conventional ssAAV vectors.

Fortunately, with sizes of the individual reprogramming factor cDNAs between 1.0 and 1.4 kb, all four are compatible with the restricted DNA packaging capacity of scAAV vectors of up to 2.4 kb. Accordingly, we engineered these to express mammalian codon-optimized (hCO) OKSM cDNAs (one factor per vector) from the cytomegalovirus (CMV) or the spleen focus-forming virus (SFFV) promoter (Supplementary Fig. 1). We were not concerned about having to use four individual AAV vectors because of ample evidence that mammalian cells can be readily co-transduced with multiple AAVs in vitro and in vivo^43^. As additional proof, we documented highly efficient co-expression of four distinct transgenes encoded by four different AAV vectors in various cells, including primary mouse hepatocytes (Supplementary Fig. 2).

Vector functionality and efficiency were first evaluated in vitro in mouse embryonic fibroblasts (MEF), a well-established model system for cellular reprogramming. Therefore, we harnessed a unique feature of the AAV system, i.e., the ability to cross-package a given vector genome into any AAV capsid—natural or synthetic—that mediates optimal gene transfer into a specific target cell. Hence, for gene delivery into MEF, we first screened a collection of synthetic AAV capsids expressing a YFP (yellow fluorescent protein) reporter and identified one as most efficient in these cells, AAV-DJ^44^ (a chimera of AAV serotypes 2, 8, and 9; Supplementary Fig. 3a). We then packaged the OKSM cDNAs into this capsid and found that both promoters yielded similar numbers of transgene-expressing MEF after transduction (Supplementary Fig. 3b). However, intracellular expression levels were higher with the SFFV promoter as compared to CMV, making it our preferred choice.

We note that use of the AAV-DJ capsid resulted in strong expression of reprogramming factors in over 80% of all cells, with no evidence for cellular toxicity (Supplementary Fig. 3b). This confirms our initial data with the YFP reporter (Supplementary Fig. 3a) and illustrates the benefits of the AAV vector system, considering that MEF are very hard to transfect with standard reagents, whose efficiencies rarely exceed 20% and which are frequently cytotoxic^45^.

**Fig. 1:**
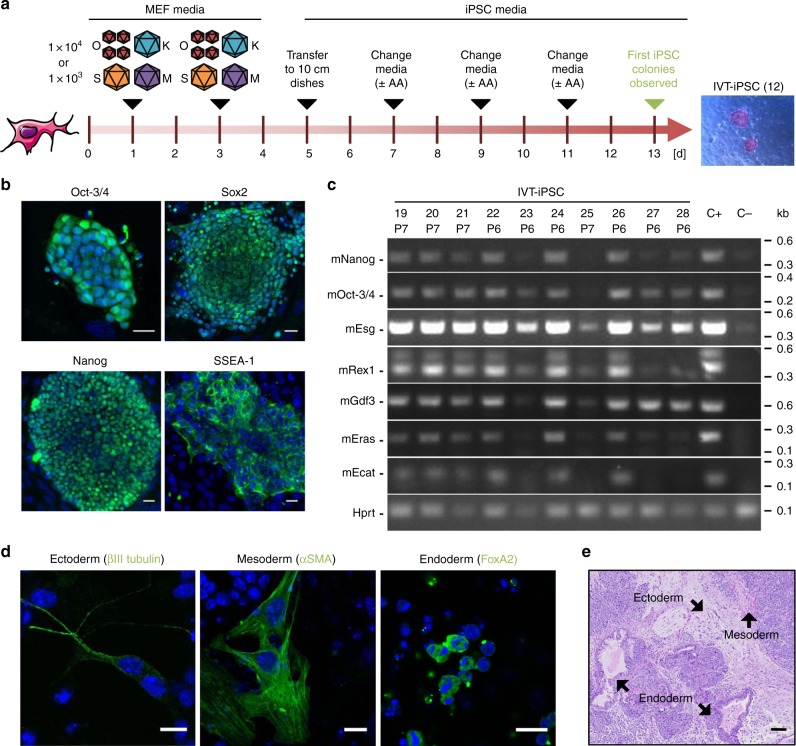
In vitro reprogramming of MEF using AAV-DJ SFFV-hCO-O/K/S/M vectors. **a** Experimental setup. MEF were transduced at days 1 and 3 after plating with the four vectors, using a 4:1:1:1 O:K:S:M stoichiometry and one of the two indicated MOIs per experiment and per vector (with a four-fold excess of Oct-3/4). At day 5, cells were transferred from a well of a 6-well plate to a 10 cm dish. Subsequently, medium was changed every other day to iPSC medium with or without ascorbic acid (AA). First colonies with iPSC morphology were observed at day 13. The image shows a representative alkaline phosphatase staining of clone IVT-iPSC 12 at passage 1. *n* = 4 for MOI 1 × 10^4^ and *n* = 3 for MOI 1 × 10^3^. **b** Confocal images exemplifying expression of Oct-3/4, Sox2, Nanog and SSEA-1 in AAV-derived iPSC (again IVT-iPSC 12). Shown are merges of Hoechst and secondary antibody (Alexa Fluor 488-labeled) stainings. Scale bars = 20 µm. **c** Reverse transcription-PCR analysis to detect expression of the depicted pluripotency markers. Shown are 10 representative clones from the reprogramming series with ascorbic acid. See Supplementary Data 1 (tab “In vitro”) for details. Six of the ten shown clones (IVT-iPSC 19 to 22, 24, and 26) are positive for all markers. Hprt (Hypoxanthine-guanine phosphoribosyltransferase) served as housekeeper. C+ iPSC generated with a lentiviral vector encoding human codon-optimized OKSM (positive control); C− untreated MEF (negative control); P, passage number. **d** Confocal images showing expression of ectodermal, mesodermal, and endodermal markers in differentiated clone IVT-iPSC 1. Shown are merges of Hoechst and secondary antibody (Alexa Fluor 488-labeled) stainings. Scale bars = 13 µm. Confocal images in **b** and **d** were taken with a Leica SP5 microscope with a ×40 oil immersion objective and processed with ImageJ. **e** Hematoxylin/eosin staining of a section of a subcutaneous teratoma generated in mice via injection of clone IVT-iPSC 1. Structures belonging to the three embryonic germ layers are indicated by black arrows. Scale bar = 100 µm. This figure contains elements from Servier Medical Art (http://smart.servier.com)

Additionally, we tested various experimental conditions including vector stoichiometry and doses (Supplementary Fig. 4 and Supplementary Data 1, tab “In vitro”), with the best giving 0.2% reprogramming efficiency and taking 13 days to yield cellular colonies (Fig. 1a). To assess their pluripotency status, we evaluated typical stem cell markers via immunofluorescence and/or reverse transcription- (RT-)PCR (Fig. 1b, c; further examples are shown in Supplementary Fig. 5). Of the 22 clones analyzed, 13 scored positive for all pluripotency markers in both assays (Supplementary Table 1), while the other nine were probably partially reprogrammed. Clones expressing the full set of markers exhibited high in vitro differentiation capacity, as shown by embryoid body formation and stainings of endodermal, mesodermal or ectodermal structures (Fig. 1d). As additional evidence, we noted spontaneous differentiation of iPSC (when cultured without LIF) into beating cells, most likely cardiomyocytes (Supplementary Movies 1, 2). Finally, we injected two iPSC clones (IVT-iPSC 1 and 12, Supplementary Data 1, tab “In vitro”) into the flanks of nude mice (each clone was injected four times) and observed formation of teratomas (cell masses originating from uncontrolled proliferation and differentiation of pluripotent cells), proving the pluripotency of the AAV-derived iPSC lines (Fig. 1e).

### Highly potent in vivo reprogramming with scAAV-OKSM vectors

**Fig. 2:**
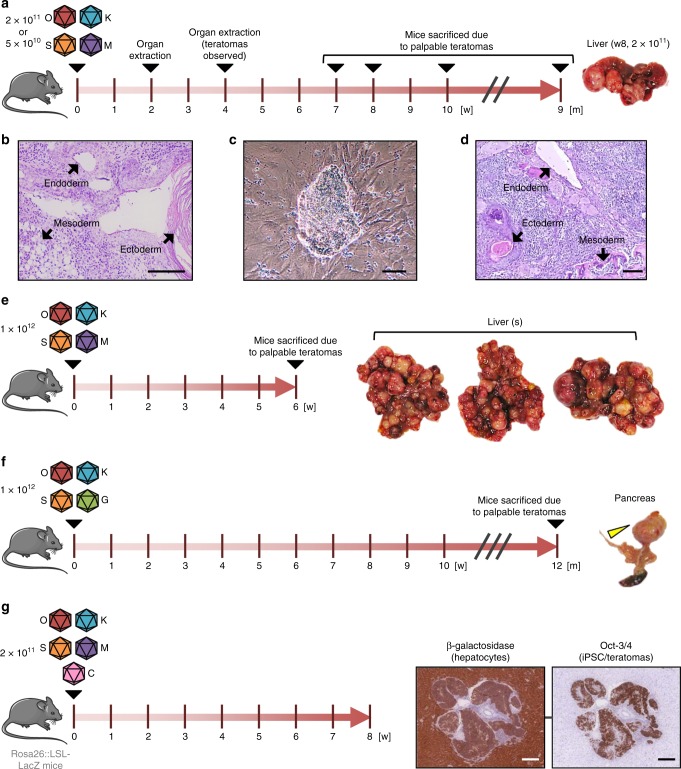
In vivo reprogramming in mice using scAAV8 SFFV-hCO-O/K/S/M vectors. **a** Experimental setup. 38 weeks-old C57BL/6 male mice were injected with 5 × 10^10^ (*n* = 9) or 2 × 10^11^ (*n* = 9) vg per vector and mouse. 2 and 4 weeks post-injection, three mice per dose and time point were sacrificed, and their organs were extracted and analyzed histopathologically. Remaining mice were sacrificed upon appearance of palpable teratomas (week [w] 7, 8 and 10: 2 × 10^11^ vg group; month [m] 9: 5 × 10^10^ vg group). **b** Hematoxylin/eosin staining of liver teratoma section (mouse treated with 2 × 10^11^ vg and sacrificed at week 8 post-injection). Structures belonging to the three embryonic germ layers are indicated by black arrows. Scale bar = 100 µm. **c** iPSC clone derived from a teratoma formed in the liver of the mouse from panel a. Scale bar = 100 µm. The shown clone is representative of 14 IVV-iPSC clones that were derived from mice treated with the OKSM cocktail (four more [IVV-iPSC 15 to 18] were later obtained with OKS, Supplementary Data 1), six of which were from teratomas (see also Fig. 3b). **d** Hematoxylin/eosin staining of a section of a subcutaneous teratoma generated in mice via injection of clone IVV-iPSC 9. Structures belonging to the three embryonic germ layers are indicated by black arrows. Scale bar = 100 µm. **e** In vivo reprogramming experiment using a high vector dose (1 × 10^12^ vg per vector and mouse) injected into 15 weeks-old C57BL/6 male mice (*n* = 3). Mice were sacrificed upon appearance of palpable teratomas at week 6 post-injection. **f** Repeat of experiment from panel **e**, using a scAAV8 vector encoding GFP instead of c-Myc (*n* = 3). All three mice were sacrificed at month 12 post-injection. One mouse (Supplementary Table 5, mouse #1, assay #6) had a teratoma (yellow arrowhead) in the pancreas. **g** Histological sections of teratomas (extracted 8 weeks post-injection) from mice injected with a cocktail of scAAV8 vectors encoding hCO-O/K/S/M or TTR promoter-driven Cre recombinase (*n* = 3). Sections were stained with hematoxylin/eosin and an anti-β-galactosidase (left) or anti-Oct-3/4 antibody (right). Scale bars = 200 µm. This figure contains elements from Servier Medical Art (http://smart.servier.com)

We next produced the scAAV-SFFV-OKSM vectors in large scale and high purity, to assess their ability to reprogram cells in adult mice. Therefore, we again harnessed the pseudotyping capability of the AAV system and now cross-packaged all four vector genomes into the AAV serotype 8 (AAV8) capsid, due to its high efficiency and broad cell specificity in vivo^46^. In a pilot experiment (Fig. 2a), we injected 18 C57BL/6 mice intravenously with two different doses (nine mice each), i.e., 5 × 10^10^ or 2 × 10^11^ vector genomes (vg) `per vector and mouse. We then sacrificed three mice per dose 2 or 4 weeks later for analyses of OKSM expression and histopathological changes.

**Fig. 3:**
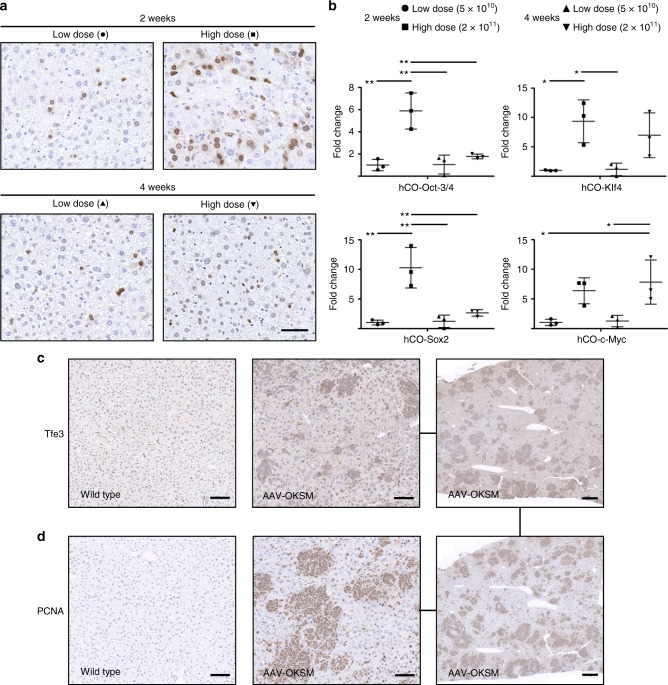
Expression of the four reprogramming factors and evidence for reprogramming in mouse liver. **a** Detection of AAV-encoded hCO-Oct-3/4 (brown staining) via immunohistochemistry in liver sections obtained from mice 2 or 4 weeks post-injection with 5 × 10^10^ (low dose) or 2 × 10^11^ (high dose) vg per vector and mouse (*n* = 3 per dose and time point). Symbols are the same as in **b**. Nuclei were counterstained with hematoxylin. Scale bar = 100 µm. **b** Quantification of the expression of AAV-encoded hCO-Oct-3/4, -Klf4, -Sox2 and -c-Myc by qRT-PCR. A delta Ct analysis was used in which the values were normalized to the expression of a housekeeper gene (actin) and then expressed as fold-changes over the values (set to 1) for the low dose at 2 weeks for each reprogramming factor. Each point represents a single mouse. Center values are means and error bars are S.D. Statistical analyses were performed by one-way ANOVA with Tukey’s Multiple Comparison Test. *, *p* < 0.05; **, *p* < 0.01. **c**, **d** Evidence for reprogramming in livers of AAV-OKSM-treated mice (2 × 10^11^ vg, sacrificed 8 weeks after administration) obtained via immunohistochemical detection of expression of Tfe3 (**c**), a marker for pluripotency, and PCNA (**d**), a marker for proliferation. Note the largely overlapping patches of Tfe3- and PCNA-positive cells in the serial sections from the same liver of an AAV-OKSM-treated mouse in the rightmost panels. Scale bars in the left and central panels = 100 µm, and in the right panels = 500 µm

As expected, we observed a robust, dose-dependent expression of all four reprogramming factors in the liver, the predominant AAV8 target from peripheral delivery at the doses that we used (Fig. 3a, b). With the exception of c-Myc, the average levels of these factors declined from week two to four, indicative of silencing of the ectopic expression cassettes as one would expect during successful reprogramming. Moreover, we found numerous patches in the livers of these animals that stained positive for Tfe3 and PCNA, markers for cellular pluripotency and proliferation, respectively (Fig. 3c, d)^47^. Besides, we also detected ectopic Oct-3/4 expression in various other tissues and cell types, such as alveolar epithelial cells in the lung, fibroblasts in the heart, or cells in the *lamina propria* in the gut (examples for one dose and one time point are shown in Supplementary Fig. 6). These observations are well in line with the known biodistribution of AAV8 in mice^46^ and validate our rationale for selecting this promiscuous serotype for our proof-of-concept study.

Congruent with this, a comprehensive histopathological analysis conducted by a trained pathologist revealed increased proliferation and nuclear pleomorphism in multiple organs at both time points (Supplementary Tables 2–4). Most notably, we found teratomas in livers of mice treated with the high dose (2 × 10^11^ vg) at 4 weeks post-injection (Supplementary Fig. 7a left). Remaining mice were thus kept alive and monitored until teratomas became palpable (summarized in Supplementary Table 5). Indeed, at week 7, 8 or 10 post-injection, all mice treated with the higher vector dose had large teratomas in the liver (Fig. 2a and Supplementary Fig. 7a). Histological analysis revealed structures belonging to the three embryonic germ layers (Fig. 2b), verifying that these were bona fide teratomas. Furthermore, we could derive iPSC lines from teratomas, blood and bone marrow from these animals (Supplementary Table 5, exemplified in Fig. 2c). Injection of two teratoma-derived iPSC clones (IVV-iPSC 9 and 14, Supplementary Data 1, tab “In vivo”) into the flanks of nude mice (each clone was injected four times) resulted in new teratomas, confirming that the AAV-derived in vivo iPSC were pluripotent (Fig. 2d). Although the liver was the organ with the highest incidence of teratomas (in line with our OKSM expression and biodistribution data in Fig. 3), we also observed extrahepatic teratomas in other tissues, including lung and thymus (Supplementary Fig. 7a and Supplementary Table 5). Finally, one mouse treated with the low dose (5 × 10^10^ vg) and sacrificed 9 months post-injection had a teratoma in the abdominal cavity arising from the pancreas or mesenteric lymph nodes (Supplementary Fig. 7b). The fact that this mouse showed no signs of reprogramming in the liver substantiates that scAAV8 vector-mediated in vivo reprogramming can also be achieved in other tissues.

### In vivo reprogramming with AAV depends on dose but not c-Myc

We subsequently injected two new mouse cohorts with an even higher dose of 1 × 10^12^ vg per vector and animal; moreover, in one cohort, we replaced the c-Myc vector with one encoding GFP (Fig. 2e, f). As a control, four mice were injected with our previous high dose of 2 × 10^11^ vg per mouse. They all had to be sacrificed due to palpable teratomas at weeks 7, 8, and 13 post-injection, confirming our findings from the pilot study in Fig. 2a (Supplementary Table 5 and Supplementary Fig. 7c). The three mice injected with the 1 × 10^12^ vg OKSM dose had to be sacrificed even earlier (6 weeks post-injection) due to massive teratoma formation in the liver (Fig. 2e) and in other organs (Supplementary Table 5), illustrating that the efficiency and kinetics of AAV-mediated in vivo reprogramming are dose-dependent.

Notably, one mouse treated with the high OKS vector dose without c-Myc had a teratoma in the pancreas at month 12 post-injection (Fig. 2f). Together with our successful isolation of iPSCs from this teratoma (clone IVV-iPSC 15 in Supplementary Fig. 11 to 12 below), this shows that in vivo reprogramming is possible even without the c-Myc oncogene.

### Cellular origin of AAV-OKSM vector-induced teratomas

To trace the cellular origin of the liver teratomas, we injected three LSL-LacZ (*loxP*-stop-*loxP*) reporter mice with 2 × 10^11^ vg of the scAAV8-OKSM cocktail per mouse, together with the same dose of scAAV8 expressing Cre recombinase from the hepatocyte-specific transthyretin (TTR) promoter^48^. At 7 to 8 weeks post-injection, nearly 100% of hepatocytes were β-galactosidase-positive (Fig. 2g, left histology panel), validating the lineage tracing. Unlike the liver, teratomas displayed variable staining, ranging from fully positive (Fig. 2g, right histology panel), to mosaic or mostly negative (Supplementary Fig. 8). Furthermore, some of the extra-hepatic masses of undifferentiated cells were also β-galactosidase-positive, reflecting the hepatic origin of these cells, which can migrate and colonize other tissues (Supplementary Fig. 9). We thus conclude that (i) hepatocytes can give rise to teratomas and that (ii) some of the observed teratomas had a polyclonal origin.

### Analysis of AAV-OKSM vector integration events

Originally, we had selected AAV vectors for in vivo OKSM delivery owing to their remarkable biosafety, illustrated by the lack of serious adverse events in over 160 clinical trials^34,35^. Corroborating this are studies of AAV vector integration sites (IS) in mice, non-human primates (NHP) and muscle or liver biopsies from vector-treated human patients^49–52^, which revealed a genome-wide, random, safe and low-frequency distribution of IS (up to 1 × 10^−3^ per cell on average in human livers) without evidence of genotoxicity. Still, a 2015 study has sparked a controversial debate on whether integration of wild-type AAV2 DNA is correlated with human liver cancers^34,35,53,54^. Besides, integration events were implied in the two previous studies that used AAV/OKSM vectors to reprogram mouse cells in vitro, but neither the consequences nor the IS were characterized^36,37^.

**Fig. 4:**
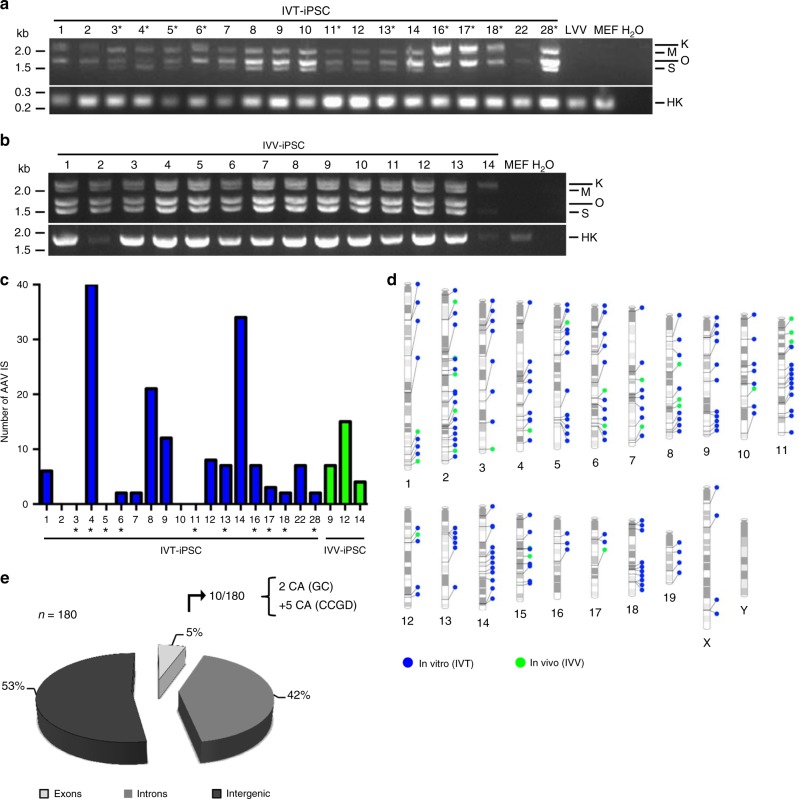
Analysis of AAV vector traces and integration sites in scAAV/OKSM-derived cellular clones. **a** PCR analysis of 19 clones generated in vitro and analyzed at an early passage (between passage 0 and 6). Top gel: PCR with primers amplifying the whole cassette, from SFFV promoter to polyadenylation signal. Three or four distinct bands are detected, which correspond to the four AAV reprogramming vectors used. Bottom gel: PCR to detect the *Hprt* housekeeper (HK) gene. LVV, iPSC cells generated with a lentiviral vector encoding human codon-optimized OKSM; MEF, untreated MEF; H_2_O, control PCR using water instead of DNA template. Sizes of a DNA marker are shown in kilobases (kb) on the left. The asterisks highlight IVT-iPSC clones which may not have been fully reprogrammed (Supplementary Data 1). **b** PCR analysis of 14 AAV-iPSC clones generated in vivo using a MOI of 2 × 10^11^ and analyzed at an early passage (between passage 2 and 4). See panel a for details as well as Supplementary Data 1 for information on all clones. **c** Graph showing the number of AAV insertion sites (IS) identified by nrLAM-PCR or TES in the cellular clones shown in **a** (blue bars, generated in vitro) and **b** (green bars, generated in vivo). **d** PhenoGram plot (generated with the tool http://visualization.ritchielab.psu.edu/phenograms/plot) depicting the distribution of AAV IS across all mouse chromosomes, segregated into cellular clones generated in vitro (blue) or in vivo (green) (same color scheme as in **c**). **e** Pie chart illustrating the proportions of AAV vector integrations (total *n* = 180) in exons, introns or intergenic regions. Of the 10 hits in exons, two affected cancer-associated (CA) genes based on the Genecards (GC) database, or seven based on the Candidate Cancer Gene Database (CCGD) (see Supplementary Data 2 for details)

Therefore, it was interesting to study whether our own AAV/OKSM-derived cellular clones carried vector integrations as well. Indeed, standard PCRs confirmed AAV vector persistence in all 33 clones analyzed (19 IVT-iPSC and 14 IVV-iPSC clones; Fig. 4a, b). To ascertain whether the genomes were integrated and, if so, to identify the precise genomic IS, we performed non-restrictive linear amplification-mediated PCR (nrLAM-PCR)^55^ and target-enrichment sequencing (TES) on a subset of 22 clones. This allowed us to define a total of 180 AAV IS in three quarters of the in vitro-derived clones and in all of those generated in vivo (Fig. 4c, Supplementary Data 2 and Supplementary Fig. 10). Numbers of IS per clone varied from 2 to 40 (Fig. 4c and Supplementary Data 2), consistent with data from AAV vector-treated humans (Supplementary Note 1). Integrations were found in clones that were partially or fully reprogrammed (Supplementary Data 1), implying they occur early during dedifferentiation. Integrated vectors were scattered over all chromosomes without hotspots (Fig. 4d and Supplementary Data 2), in line with the random AAV vector integration profiles in mice, NHPs and humans^49–51^, and arguing against clonal selection of AAV IS in vitro or in vivo. Of the 180 IS, 85 (47.23%) were within genes (Fig. 4e, sum of exons and introns), congruent with reported values in mice between 41 and 72%^50,56^. Importantly, only 10 IS (5.56%) were in exons, and of these, only two (1.11% of total 180 hits) or seven (3.89%) affected genes that are associated with cancer (numbers depend on the cancer gene database that was queried, see Supplementary Data 2 and Supplementary Note 1 for more details). Notably, we observed no genuine tumors in any of our mice, not even 9 months or later after OKS(M) vector injection. The single exception is a mediastinal lymphoma in a 84 week-old mouse that was, however, most likely age- and not vector-related (Supplementary Table 4). As a whole, our data are thus congruent with the bulk of prior studies that found no evidence for enhanced tumorigenesis in animals or humans after AAV vector delivery and integration^51^. Besides, as expected for properly reprogrammed iPSC, integrated ectopic OKSM vector genomes were mostly transcriptionally silent, while endogenous pluripotency markers were strongly induced (Supplementary Fig. 11).

## Discussion

Here, we aimed to remove a major roadblock that still hampers the broader use of the exciting concept of in vivo reprogramming and ultimately its clinical translation, namely, the lack of OKSM delivery systems that (i) function in any mammalian species and are independent of transgenic animals, (ii) can be targeted to any desired cell or organ within an intact organism, (iii) are compatible with tissue-specific and inducible promoters for additional layers of control, and (iv) do not rely on complicated administration schemes that are hard or impossible to apply in humans. While all of the previously reported OKSM delivery tools meet a subset of these demands, none of them affords the juxtaposition of specificity and versatility that is required to take the field of in vivo reprogramming to the next level. This combination of key assets is provided, however, by recombinant AAV vectors, as evidenced in the present work and supported by a wealth of preclinical and clinical data from the past three decades, including the recent commercialization of AAV-based gene therapy products (Glybera, Luxturna).

Indeed, the data presented here demonstrate that when appropriately pseudotyped, scAAV are very potent reagents for in vitro and in vivo somatic cell reprogramming. In cultured cells, the chimeric capsid AAV-DJ yielded robust reprogramming efficiencies of 0.1 and 0.2% from low multiplicities of infection (MOIs) of 1 × 10^3^ or 1 × 10^4^, respectively. This compares favorably to ssAAVs^37^, which gave 0.001 to 0.091% at MOIs of 2 × 10^4^ to 2 × 10^5^, respectively, and no iPSC colonies at 2 × 10^3^. Our lowest MOI was also three orders of magnitude lower (1 × 10^3^ vs. 1 × 10^6^), and our reprogramming efficiencies higher (0.1% vs. 0.036%), than those of an OKS-encoding ssAAV2 triple-tyrosine mutant in mouse adipose stromal cells^36^, which are very amenable to reprogramming^57^. The likely reason for the superior performance of our vectors is the unique combination of (i) potent capsid, (ii) strong promoter, (iii) scAAV genome, and (iv) codon-optimized OKSM cDNAs.

These factors also explain their high in vivo efficiency, evidenced by the appearance of bona fide teratomas in multiple organs, even without c-Myc, circulating iPSC, and dose-dependent increases in mitosis (indicative of early in situ reprogramming events). Teratomas are a most stringent endpoint of in vivo reprogramming and are typically noted in OKSM-transgenic mice^5,6^, exemplifying the ability of our vectors to phenocopy this key readout from a simple and single peripheral low-pressure administration.

Based on our proof-of-principle work, we envision numerous applications of scAAV/OKS(M) vectors for cellular reprogramming in animals and, ultimately, humans. While teratomas—albeit poorly invasive and poorly metastatic—and morbidity are obviously undesirable clinical outcomes in patients and need to be avoided, data in OKSM-transgenic mice show that these outcomes depend on the extent and length of OKSM expression^5,8^. The next vector generation should thus capitalize on the vast experience with chemically and/or physically regulatable promoters in AAV vectors^.^^58–60^, and exploit these to tightly control in vivo OKS(M) expression. This should ideally allow to recapitulate the concept of curtailed reprogramming via short-term, cyclic or pulsed OKSM expression^8,14–17^, yielding partially dedifferentiated cells that no longer form tumors in vivo while remaining capable of somatic differentiation. To this end, we propose a second generation of scAAV vectors in which the duration of OKSM expression is, e.g., stringently controlled by light or which can be turned off at any given time by means of CRISPR cleavage. While the present study already shows that AAV-mediated ectopic OKSM expression is inherently silenced over time (Fig. 3), as expected during reprogramming, these or other newly introduced control mechanisms should allow to accelerate this process and thus further reduce the risk of in situ teratoma formation.

Other complementary safety measures should include modulation of vector dose, to additionally limit intracellular OKSM expression and to concurrently minimize undesired transduction of off-target organs. In this respect, we consider it highly beneficial that our vectors express each factor individually, as it facilitates fine-tuning of OKS(M) doses and ratios which may be of value for partial reprogramming strategies^17^. Here, the dose dependency of in vivo reprogramming efficiency, specificity and safety with scAAV8-OKSM vectors was clearly illustrated by the different outcomes observed at our doses of 5 × 10^10^, 2 × 10^11^ or 1 × 10^12^ vg per vector and mouse, i.e., 20-fold dose range. Our findings on dose-dependent AAV8 promiscuity in mice are well in line with previous data, e.g., from the Kay group who noted a strong liver tropism in animals injected with 3 × 10^11^ vg (comparable to our dose of 2 × 10^11^ and the ensuing results), but a very broad transduction in other tissues at 7 × 10^12^ vg^46^ (see also Supplementary Fig. 7c).

Consequently, a rewarding additional and complementary measure to enhance specificity and safety of future AAV-OKSM iterations will be to select from the wealth of AAV capsids and promoters with desired biodistribution (here, the broadly active AAV8 and SFFV were merely used for proof-of-concept), and to also combine this with miRNA-based detargeting strategies^61^. Importantly, unlike all other viral or non-viral vector systems that have been used for in vivo OKSM delivery before, this will offer the option to target OKSM expression to virtually any organ of choice, following a simple and single intravenous administration. This is owing to the high amenability of the AAV capsid and genome to genetic diversification^62^, permitting to apply a wide portfolio of molecular evolution technologies and to select superior capsids in any desired tissue, as already demonstrated extensively in liver^44^, brain^63^, eye^64^, heart^65^, ear^66^ or skeletal muscle^67^, among many others. Most recently, by high-throughput in vivo screening of comprehensive AAV capsid libraries, we have also identified synthetic AAV capsids that outperform the best wild-types in skeletal muscle, heart and diaphragm, or that mediate highly selective transgene expression in specific cell types within the murine liver (unpublished data).

We are thus highly optimistic that these and other modifications will ultimately provide sufficient control over in vivo OKSM delivery and expression, and thereby not only enable a broader use of in vivo reprogramming technology in any desired animal model, but also facilitate and accelerate its application for therapeutic reprogramming. Already, a rapidly increasing number of reports showcase the tremendous potential of direct in vivo reprogramming in multiple tissues such as liver, muscle or brain, including repair and functional recovery after tissue damage, amelioration of age-related phenotypes as well as increased resistance to metabolic disease in mice^7,8,19–23^. Nonetheless, these previous studies and associated commentaries^24–27^ also consistently pointed out the need for better, clinically acceptable OKSM delivery means, a critical gap that may be closed in the future based on the AAV-OKSM vectors reported here.

Importantly, this may then also help to alleviate concerns with alternative strategies such as transplantation of ex vivo differentiated iPSC or embryonic stem cells, which often fail to mature completely, trigger immune rejection (in the case of allogenic or xenogenic cells) and/or do not engraft and survive long-term^22,23,27,68^. Partial or transient in vivo reprogramming may also be beneficial over lineage conversion approaches as these do not allow for expansion of the transdifferentiated cells, and efficiencies are typically low^2^. In contrast, controlled, vector-mediated in vivo dedifferentiation of cells towards plastic intermediates will yield mitotically active progenitor populations with high multi-lineage differentiation capacity and thus numerous medical implications. Last but not least, in vivo generated stem cells could represent a new source of autologous cells that alleviates constraints of cell culture-derived iPSC, such as ex vivo GMP requirements, pathogen contamination or concerns about iPSC antigenicity^69^.

Still, before these concepts can progress to clinical application in humans, safety and other lasting repercussions of introducing potent reprogramming factors in vivo will have to be monitored prudently, also in larger animal models. Next to the aforementioned risk of uncontrolled proliferation and teratoma formation, one parameter that requires particular attention is the propensity of cells to stably integrate AAV vector DNA during dedifferentiation, as noted before and here in vitro^36,37^, and as further observed by us in the present work in vivo. Most importantly, the frequencies and sites of AAV vector integration detected in the current study are highly similar to those reported^50^ in biopsies from humans who had received Glybera, a therapeutic AAV1 vector that was granted marketing authorization by the European Commission in 2012. Combined with our notions of very rare integration into exons of cancer-associated genes and the complete absence of signs of tumorigenesis in our mice, we thus carefully conclude that the use of AAV/OKS(M) vectors for direct in vivo reprogramming may be safe from a genotoxicity standpoint, or at least no more hazardous than any conventional AAV gene transfer/therapy (see also Supplementary Note 1).

## Methods

### Cell culture

All cell lines and primary cells were incubated in a humidified atmosphere at 37 °C and 5% CO_2_. In general, cells were passaged two or three times a week by washing them once with sterile 1 × PBS (Life Technologies GmbH, Paisley, UK), incubating with 0.25% trypsin/EDTA (Life Technologies GmbH) for 5 (HEK293T cells^70^, CRL-3216, ATCC, Manassas, VA, USA) to 10 min (mouse embryonic fibroblasts, MEF), re-suspending the trypsinized cells in fresh medium (DMEM (Life Technologies GmbH), 10% FBS (Biochrom, Cambourne, UK) and 1% penicillin-streptomycin (Life Technologies GmbH)) and re-seeding them at the desired density.

Mouse embryonic stem cells (mESC E14TG2α^71^, CRL-1821, ATCC) were cultured in gelatin-coated dishes with Knockout^TM^ DMEM supplemented with 15% Knockout^TM^ Serum Replacement, 1% penicillin-streptomycin, 1% L-glutamine, 1% Non-essential Amino Acid Solution, 50 µM β-mercaptoethanol (all Life Technologies GmbH) and 5 × 10^5^ units LIF (Merck Millipore, Darmstadt, Germany). They were passaged as described above two or three times a week on demand.

For the reprogramming experiments, primary MEF from C57BL/6 mice (kind gift from Annabel Grewenig, German Cancer Research Center, Heidelberg, Germany) at passage 3 were seeded in 6-well plates (Greiner Bio-One, Frickenhausen, Germany) coated with gelatin (Sigma-Aldrich, Munich, Germany) at a density of 1 × 10^5^ cells per well (day 0). The next day (day 1), the cells were transduced with the reprogramming AAV vectors or, as control, 5 µl of a crude lysate of the lentiviral vector pRRL.PPT.SF.hOKSMco-idTom-pre-FRT (kind gift from Axel Schambach, Hannover Medical School, Germany). The multiplicity of infection (MOI) for each AAV vector was 1 × 10^3^ or 1 × 10^4^ viral genomes (vg) per cell, except for AAV-DJ SFFV-hCO-Oct-3/4 for which 4 × 10^3^ or 4 × 10^4^ vg per cell were used (corresponding to a O:K:S:M stoichiometry of 4:1:1:1). Cells were then centrifuged at 1231 × *g* for 15 min. 2 days later (day 3), the medium was changed to mESC medium (see above), and the cells were transduced again as described above. After 2 days (day 5), the cells from each well of the 6-well plate were transferred to gelatin-coated 10 cm dishes. After this point, the medium (in some cases supplemented with ascorbic acid to a final concentration of 50 mg/ml) was changed every other day until iPSC colonies were observed. Single colonies were picked and transferred to gelatin-coated 12-well plates with feeder layers.

To prepare the latter, MEF were grown to 80% confluency in T75 cell culture flasks (Greiner Bio-One). One ml of medium was then removed and 1 ml mitomycin C (Sigma-Aldrich) was added to a final concentration of 10 µg/ml. The cells were incubated for 2 h at 37 °C and 5% CO_2_. Then, they were washed twice with sterile 1 × PBS, trypsinized, counted and seeded in gelatin-coated 6-well plates at a density of 2.5 × 10^5^ cells per well. To passage iPSC, the medium (iPSC medium plus 2i, i.e., 1 µM PD0325901 and 3 µM CHIR99021 (Axon Medchem, Groningen, Netherlands)) was removed, the cells were washed with 2 ml sterile 1 × PBS and incubated with 500 µl 0.25% trypsin/EDTA for approximately 10 min at 37 °C and 5% CO_2_. The trypsinized cells were then collected with 1 ml sterile 1 × PBS and trypsin was inactivated by adding 1 ml medium. The cells were centrifuged for 5 min at 335 × *g*, resuspended in 8 ml fresh medium and re-added to the 6-well plates. To separate the feeder layers from the iPSC, the cell suspension was incubated for 2 h at 37 °C and 5% CO_2_. Then, the supernatant containing the iPSC was harvested and re-seeded at the desired density on gelatin-coated dishes with fresh feeder layers.

To differentiate iPSC into the three embryonic germ layers, embryoid bodies (EB) were prepared using hanging drops. To this end, iPSC were passaged as described above and diluted in EB medium (Knockout^TM^ DMEM, 15% Knockout^TM^ Serum Replacement, 1% L-glutamine, 1% Non-essential Amino Acid Solution) to a density of 600 cells per 20 µl (or 3 × 10^4^ cells per ml). Using a multichannel pipette, 70 to 100 drops of 20 µl each were deposited onto the lid of a bacterial petri dish (Greiner Bio-One). Then, 20 ml of sterile 1 × PBS were poured into the petri dish to maintain humidity, and the dish was closed by carefully placing the drop-containing lid on top. The hanging drops were incubated for 3 days at 37 °C and 5% CO_2_. Afterwards, they were harvested with 4 ml EB medium per lid (three lids containing hanging drops were combined into a single petri dish) and cultured in suspension for three additional days in 10 cm dishes. Next, the EB were transferred to adherent culture. For this purpose, they were harvested into their own medium, transferred to a conical tube, let sediment for around 10 min at room temperature, resuspended in fresh EB medium and transferred to gelatin-coated black µ-Plate 96-well plate (Ibidi, Martinsried, Germany), aiming to obtain one to two EB per well. The EB were incubated at 37 °C and 5% CO_2_ for 1 to 2 weeks. During this period, medium was changed every other day, and EB were monitored regularly for signs of differentiation. Once differentiated structures were observed, the EB were stained with antibodies against markers of endoderm, mesoderm or ectoderm, following the immunostaining protocol described below.

### AAV vector production and titration

AAV vectors for capsid screening were produced in small scale by triple-transfecting HEK293T cells using polyethylenimine (PEI) with (i) an AAV vector plasmid, (ii) an AAV helper plasmid expressing *rep* and *cap*, and (iii) an adenoviral helper plasmid^72^. The capsids used were AAV-DJ^44^, AAV-DJ with a tyrosine-to-phenylalanine mutation of tyrosine 500 (equivalent position in the AAV-2 capsid gene, Y500F^73,74^), AAV2 with tyrosine-to-phenylalanine mutations of tyrosines 444 and 500 (Y444 + 500F) or of tyrosines 500 and 730 (Y500 + 730 F)^73,74^, and AAV1 with insertions of peptides NDVRSAN (P4) or NDVRAVS (P5) into an exposed capsid region (between AAV1 residues D590 and P591).

For in vitro experiments, AAV vectors were produced in medium scale and purified through iodixanol gradient density centrifugation, and full AAV particles were collected from the 40% iodixanol phase^75^.

For in vivo experiments, AAV vectors were produced in large scale, by triple-transfecting 30 15 cm dishes using PEI^72,75^ and purifying resulting vector particles using cesium chloride (CsCl) gradient density centrifugation^44^. Samples with a refractive index between 1.3711 and 1.3766 comprising DNA-containing AAV particles were pooled and dialyzed against 1 × PBS, using Slide-A-Lyzer Dialysis cassettes (Thermo Fisher Scientific, St. Leon-Rot, Germany) according to the manufacturer’s instructions. The dialysis procedure comprised a 30 min incubation at room temperature with no stirring, followed by several periods with gentle stirring at 4 °C and replacement of 1 × PBS in between: 1, 2 h, overnight, 2, 2 h. Upon completion of the dialysis, the sample was removed from the cassette and concentrated using an Amicon^®^ Ultra Centrifugal Filter (Millipore, Billerica, MA, USA), which was previously equilibrated by washing it twice with 15 ml 1 × PBS. Several centrifugation steps at 400 × g for less than 2 min were used to reduce the volume of the sample to 1 to 1.5 ml. The purified AAV vectors were finally aliquoted and stored at −80 °C.

The AAV vectors purified with either iodixanol or CsCl gradients were titered using quantitative real-time PCR (qPCR). For this purpose, the DNA was extracted from the AAV vectors using an alkaline lysis protocol, in which 10 µl of the AAV vector sample were mixed with 10 µl TE buffer and 20 µl 2 M NaOH, followed by incubation for 30 min at 56 °C. The alkaline lysis was stopped by adding 38 µl 1 M HCl, and the final volume was adjusted to 1 ml by adding 922 µl RNase-free H_2_O. Iodixanol-purified samples were further diluted 1:10 with H_2_O to avoid interference of the iodixanol with the qPCR reaction. As a negative control, RNase-free H_2_O was used, and as a positive control an AAV vector whose titer was already known. The qPCR was performed using the SensiMix^TM^ II Probe Kit (Bioline, London, UK). Primer pairs were CMV_5′ and CMV_3′, SFFV_5′ and SFFV_3′, or TTR_5′ and TTR_3′ (see Supplementary Table 6 for all primer sequences), and probes were CMV (FAM-agtcatcgctattaccatgg-BHQ1), SFFV (FAM-acctgaaatgaccctgcgccttatttgaattaac-BHQ1) or TTR (FAM-tttggagtcagcttggcagggatca-BHQ1) (FAM, Fluorescein amidite; BHQ1, black hole quencher 1). The reaction mixes were prepared in triplicates and contained (per reaction): 1 × SensiMix II Probe Kit, 0.4 µM of each primer, 0.1 µM of the probe, 1.43 µl of the alkaline lysis reaction (see above) and RNase-free H_2_O to adjust the final volume to 10 µl. The standard curve was prepared by making serial dilutions of an appropriate plasmid, with numbers of molecules per reaction ranging from 5 × 10^3^ to 5 × 10^8^. The qPCR was performed in a Rotor-Gene Q (QIAGEN, Hilden, Germany) using the following amplification conditions: 10 min at 95 °C, followed by 40 cycles of denaturation at 95 °C for 10 s and annealing/extension at 60 °C for 20 s. The results were analyzed using the Rotor-Gene Q Series Software 1.7 (QIAGEN). A standard curve was considered reliable when its R^2^ was greater than 0.985. To calculate titers of viral genomes per ml (vg/ml), the obtained concentration was multiplied by 7 (a 10 µl reaction contained 1.43 µl of the original alkaline lysis reaction), 100 (dilution after alkaline lysis), 100 (to convert 10 µl into 1 ml) and 10 (only for samples purified with iodixanol gradients diluted 1:10 after alkaline lysis).

### Cloning procedures

The human codon-optimized OKSM cDNAs were PCR-amplified from plasmid pRRL.PPT.SF.hOKSMco-idTom-pre-FRT^76^ and cloned into a self-complementary (sc)AAV backbone using BamHI/SalI restriction sites. The SFFV promoter-containing reprogramming plasmids were cloned in two steps. Firstly, the SFFV promoter together with the human codon-optimized Oct-3/4 cDNA were PCR-amplified from lentiviral plasmid pRRL.PPT.SF.hOKSMco-idTom-pre-FRT with primers SFFV_For_EcoRI and hOct4_Rev_SalI, and cloned into the EcoRI/SalI-digested scAAV backbone. Secondly, the other three reprogramming factors were PCR-amplified with primers hKlf4_For_BamHI and hKlf4_Rev_SalI, hSox2_For_BamHI and hSox2_Rev_SalI, or hcmyc_For_BamHI and hcmyc_Rev_SalI, respectively, and cloned into the scAAV-SFFV-hCO-Oct-3/4 plasmid via BamHI/SalI (a BamHI site was present in the original pRRL.PPT.SF.hOKSMco-idTom-pre-FRT plasmid between the SFFV promoter and the Oct-3/4 cDNA) (see Supplementary Table 6 for primer sequences). The scAAV-SFFV-GFP vector was cloned by isolating the GFP cDNA from pBS-H1-TuD-empty-GFP^77^ via BamHI/SalI digestion and ligating it into BamHI/SalI-digested scAAV-SFFV-hCO-Oct-3/4.

### Reverse transcription-PCR (RT-PCR)

RT-PCR was performed to detect expression of pluripotency markers in iPSC. For this purpose, cell pellets harvested from a 6-well plate were resuspended in 1 ml QIAzol Lysis Reagent (QIAGEN). RNA was extracted using the miRNeasy Mini Kit (QIAGEN) following a supplementary protocol for purification of total and small RNA from serum or plasma (https://www.qiagen.com/us/resources/resourcedetail?id=baa5450e-739b-4a11-8006-745796a56554&lang=en; this protocol was used due to the low amount of starting material since iPSC were harvested after differential sedimentation). After resuspension in 1 ml QIAzol Lysis Reagent, the samples were incubated for 5 min at room temperature. Then, 200 µl chloroform were added to the homogenate, and the tube was closed securely and shaken vigorously for 15 s. This was followed by a 2 to 3 min incubation at room temperature and a 15 min centrifugation at 13,400 × *g* and 4 °C. Subsequently, 770 µl of the transparent upper phase containing the RNA were transferred to a new tube, and 1155 µl 100% ethanol were added and mixed thoroughly by pipetting up and down. The sample was then transferred to an RNeasy Mini spin column and centrifuged for 15 s at 9300 × *g* and room temperature. An on-column DNase I digestion was performed following the instructions in Appendix D of the miRNeasy Mini Handbook (QIAGEN). Finally, the column was washed twice with 500 µl buffer RPE, residual RPE buffer was eliminated by an additional centrifugation step, and the RNA was eluted in 30 µl RNase-free H_2_O.

Reverse transcription was performed using the Tetro cDNA Synthesis Kit (Bioline). The cDNA synthesis reaction had a total volume of 20 µl and contained 240 ng RNA, 1 µl Random Hexamer Primer Mix, 1 µl 10 mM dNTP mix, 1 µl Ribosafe RNase Inhibitor, 1 µl Tetro Reverse Transcriptase (200 U/µl) and 4 µl 5 × RT Buffer. The reaction was incubated for 10 min at 25 °C, followed by 30 min at 45 °C and 5 min at 85 °C for termination. The subsequent PCR was performed using published primers^4^. The PCR reaction contained 1 to 3 µl cDNA, 4 µl 5 × Phusion HF buffer (Thermo Fisher Scientific), 1 µl forward primer (10 nM), 1 µl reverse primer (10 nM), 0.4 µl 10 nM dNTPs (NEB, Frankfurt, Germany), 0.6 µl DMSO (Thermo Fisher Scientific), 0.2 µl Phusion Hot Start II DNA polymerase (Thermo Fisher Scientific) and H_2_O to a total volume of 20 µl. PCR conditions were initial denaturation at 98 °C for 30 s, followed by 30–35 cycles of denaturation at 98 °C for 10 s, primer annealing at 55 °C for 15 s and extension at 72 °C for 30 s. This was followed by a final extension step at 72 °C for 8 min. PCR products were separated in a 2% agarose gel with ethidium bromide and visualized with the Gel Doc XR (Bio-Rad, Munich, Germany).

### Quantitative reverse transcription PCR (qRT-PCR)

qRT-PCR was used to detect expression of the exogenous reprogramming factors and pluripotency markers in iPSC. Reverse transcription was performed using the Tetro cDNA Synthesis Kit (Bioline) as described above, but using 500 ng RNA extracted with the AllPrep DNA/RNA/miRNA Universal Kit (QIAGEN) following the manufacturer’s instructions. Each qRT-PCR reaction contained 6.25 µl Power SYBR Green PCR Master Mix (Thermo Fisher Scientific), 1.75 µl of a primer mix containing the forward and reverse primers (Supplementary Table 6) at a concentration of 10 µM, 1.25 µl cDNA and 3.25 µl H_2_O. The qRT-PCR reaction was run in an Applied Biosystems StepOnePlus Real-Time PCR system (Thermo Fisher Scientific).

To detect the expression of the exogenous reprogramming factors in vivo, organs were homogenized with the Precellys Evolution Homogenizer (Bertin Instruments, Frankfurt, Germany), using 2.8 mm zirconium oxide beads and 1 ml of TRIzol Reagent (Ambion, Life Technologies GmbH). RNA was extracted following the manufacturer’s instructions. RNA was treated with TURBO DNA-free Kit (Ambion, Life Technologies GmbH) to remove genomic DNA contamination. For cDNA synthesis, 1 µg of each RNA was used and reverse transcription was performed using 4 µl of iScript RT Supermix (Bio-Rad) in a 20 µl reaction. The reaction was incubated at 25 °C for 5 min for priming, then at 46 °C for 20 min for reverse transcription and finally at 95 °C for 1 min to inactivate the reverse transcriptase. The cDNAs were diluted 1:10 in RNase-free H_2_O. Each qRT-PCR reaction contained 5 µl of Applied Biosystems PowerUp SYBR Green Master Mix (Thermo Fisher Scientific), 2 µl of cDNA at a concentration of 5 ng/µl and 0.5 µl of each primer (forward and reverse) at a concentration of 10 µM. The qRT-PCR reaction was run in an Applied Biosystems 7900HT Real-Time PCR system (Thermo Fisher Scientific).

### Non-restrictive linear amplification-mediated PCR (nrLAM-PCR)

The nrLAM-PCR reaction^24^ consisted of a linear PCR (primers P2 and P3, Supplementary Table 6), followed by magnetic capture of the DNA. Then, the PCR products were ligated to a bar-coded linker cassette using circLigase (Epicentre, Madison, WI, USA). This was followed by two exponential PCRs (primers P5 and LCI were used in the first exponential PCR, and primers P4 and LCII in the second; Supplementary Table 6) and by a final Mega PCR in which bar-coded primers were used (MiSAAV primers and MegaLinker primer; Supplementary Table 6). To rule out a contamination of the controls, a small fraction of the Mega PCR products were run on a 2% agarose gel. The rest was purified using the Agencourt AMPure XP PCR purification system (Beckman Coulter, Brea, CA, USA) following the manufacturer’s instructions. In total 50 ng of each sample were pooled and sequenced on a MiSeq Illumina platform at the Deep Sequencing Core Facility of the German Cancer Research Center (Heidelberg, Germany).

### Target Enrichment Sequencing (TES)

Genomic DNA was extracted using the AllPrep DNA/RNA/miRNA Universal Kit (QIAGEN), and the concentration was measured in a Qubit (Life Technologies GmbH). To perform TES, 1 µg genomic DNA was fragmented with a S220 Focused-ultrasonicator (Covaris, Woburn, MA, USA) to an average peak size of about 250 bp. Libraries were prepared with the SureSelectXT2 Reagent Kits (Agilent Technologies, Waldbronn, Germany). Vector-containing fragments were enriched using custom SureSelect baits (Agilent Technologies) complementary to the AAV vector used for reprogramming (in combination with other vector baits). The libraries were submitted to the sequencing facility of the German Cancer Research Center for 100 bp paired-end sequencing on a HiSeq 2000. Insertion sites were determined with our in-house “GENE-IS” pipeline^78^.

### Immunostaining and microscopy

Alkaline phosphatase stainings were performed using the Alkaline Phosphatase Staining Kit II (Stemgent, Cambridge, MA, USA) in 24-well plates (Greiner Bio-One) following the manufacturer’s instructions. Images were taken with an AmScope microscope digital camera MD800E (AmScope, Irvine, CA, USA) attached to an Olympus CKX41 microscope (Olympus, Hamburg, Germany).

Immunostainings to detect pluripotency or differentiation markers were performed on iPSC cultured in black µ-Plate 96-well plates (Ibidi) coated with gelatin. Cells were washed twice with ice-cold 1 × PBS and fixed with 100% methanol for 7 min at −20 °C. Methanol was rinsed with acetone for 20 s at −20 °C. Once fixed, the cells were washed three times with PBST (1 × PBS, 0.1% Tween20) for 5 min at room temperature. Then, they were blocked for 30 min at room temperature with blocking buffer (1 × PBS, 0.5% BSA, 1% FCS and 0.1% Triton X-100) and incubated overnight at 4 °C with the primary antibody. Antibodies to detect pluripotency markers were anti-Oct-3/4 (1:150, sc-8628, Santa Cruz Biotechnology, Heidelberg, Germany), anti-Nanog (1:150, ab80892, Abcam, Cambridge, UK), anti-Klf4 (1:20, AF3640, R&D Systems, Wiesbaden-Nordenstadt, Germany), anti-Sox2 (1:200, AB5603, Merck), and anti-SSEA-1 (1:150, sc21702, Santa Cruz Biotechnology). Antibodies to detect differentiation markers were anti-βIII-tubulin (1:50, sc-51670, Santa Cruz Biotechnology), anti-α-smooth muscle actin (1:400, A 5228, Sigma-Aldrich), anti-FoxA2 (1:50, AF2400, R&D Systems). All antibodies were diluted in blocking buffer. Following incubation with the primary antibody, cells were washed twice with blocking buffer and incubated for 4 h at 4 °C with the secondary antibody (1:500, anti-rabbit, anti-goat or anti-mouse Alexa Fluor 488-conjugated, Life Technologies GmbH). Then, the cells were washed twice with ice-cold PBST, incubated for 3 min with 1 × PBS containing Hoechst 33258 (Life Technologies GmbH) diluted 1:3000 and washed again with ice-cold 1 × PBS. The cells were imaged and stored (at 4 °C) immersed in 1 × PBS. Images were taken with an Olympus Biosystems IX81 microscope with a 10 × objective or with a Leica SP5 confocal microscope (Leica, Mannheim, Germany) with a 40 × oil immersion objective.

### Flow cytometry

To analyze AAV vector-transduced cells by flow cytometry they were cultured and transduced in 96-well plates. To prepare them for the analysis, the media was removed, they were washed once with 1 × PBS, trypsinized with 40 µl 0.25% trypsin/EDTA for 5 to 10 min and re-suspended in 160 µl 1% BSA in 1 × PBS. Data were acquired with the Cytomics FC500MPL analyzer and the MXP software (both Beckman Coulter). First, size and granularity of the cells were determined by plotting the forward and side laser light scatter. Cells with the largest size and highest granularity were gated as “living cells”. Then, fluorescence of the living cells was analyzed by plotting the green vs. the red channel. The gate for positive cells was established by measuring an untransduced control and excluding the area from the gate in which events appeared. The untransduced control was also used to determine the appropriate voltage. The acquisition parameters were set to measure 2 × 10^4^ events or to acquire for 60 s and to mix the sample three times before acquiring. The volume used for the analysis was 100 µl.

### Cloning of fluorophore-encoding vectors

Self-complementary AAV vectors expressing a CMV promoter-driven *gfp* (dsAAV-GFP) or *yfp* cDNA (dsAAV-YFP) were kindly provided by Kathleen Börner (Heidelberg University Hospital, Heidelberg, Germany). Variants of these expressing either *mScarlet* or *mTurquoise* were subsequently generated as follows. The *mScarlet* cDNA was PCR-amplified from vector pmScarlet_C1 (kind gift from Dorus Gadella [University of Amsterdam, Netherlands]; Addgene plasmid # 85042) using primers mScarlet_fw and mScarlet_re. *mTurquoise* was PCR-amplified from vector pmTurquoise2-C1 (kind gift from Dorus Gadella; Addgene plasmid # 60560) using primers mTurquoise_fw and mTurquoise_re (see Supplementary Table 6 for primer sequences). Sequences in capital letters denote overhangs containing restriction sites for cloning, where needed. Amplicons were digested with AgeI/NheI and ligated into AgeI/NheI-linearized dsAAV-GFP, yielding vectors dsAAV-mScarlet and dsAAV-mTurquoise.

HEK293T cells (25,000 cells per compartment) and primary hepatocytes (10,000 cells per compartment) were seeded into 8-well chamber slides (Ibidi, #80826). The next day, cells were transduced with Iodixanol-purified AAV-DJ vectors encoding a CMV promoter-driven *mTurquoise*, *egfp*, *eyfp* or *mScarlet* cDNA (one per vector, see above) either separately (MOI 1 × 10^4^) or with all four viruses in combination (MOI 1 × 10^4^ each). 48 h post-infection, fluorescence was observed using a Leica SP8 confocal laser scanning microscope equipped with a UV, argon and solid state laser and a HC PL APO 40 × oil objective (N/A = 1.3).

Laser power used to excite the different fluorophores in the HEK293T and primary hepatocytes samples, respectively, was 3.5%/36.5% for the 405 nm UV laser (mTurquoise excitation); 3.7%/7.6% for the 488 nm argon laser line (EGFP excitation); 0.1%/0.1% for the 514 nm argon laser line (EYFP excitation) and 0.3%/0.9% for the solid state green laser (mScarlet excitation). Detection wavelengths were set as follows: 441 to 479 nm for mTurquoise, 491 to 505 nm for EGFP, 550 to 569 nm for EYFP, and 583–715 nm for mScarlet. Gain was set to 900 for each detector. These settings were chosen to minimize bleed-through between the four channels under the used experimental conditions. To visualize cells expressing all four fluorophores (“overlap” in Supplementary Fig. 2), a threshold was applied to each channel using ImageJ (version 1.51n; http://imagej.nih.gov/ij). Pixels that were above threshold in all four channels were then projected in white color onto the corresponding bright-field image.

### Animal experiments

Animal experimentation was performed at the CNIO (Madrid, Spain) according to the protocol (PROEX125/14) approved by the CNIO-ISCIII Ethics Committee for Research and Animal Welfare (CEIyBA). Wild-type male mice of 15 weeks (or 38 weeks in the pilot experiment) of age were retro-orbitally injected with the indicated AAV8 vector doses. For the tracing experiments, we used a GtRosa26^tm1Sor^ (R26R) reporter transgenic mouse model^79^. All mice used were in a pure C57BL/6 J.Ola.Hsd genetic background. Mice were monitored and sacrificed at the indicated time points or when teratomas were palpable. For subcutaneous teratomas, iPSCs were trypsinized and 1 × 10^6^ cells, suspended in iPSC medium, were subcutaneously injected into the flanks of immunocompromised nude mice (Hsd: athymic Nude/Nude from Harlan Ibérica (now Envigo, Valencia, Spain)). iPSC cells had been previously cultured in 2i medium (see above) for at least 10 days. Teratomas were isolated when the diameter reached at least 1.5 cm and processed for histological analysis.

### Isolation of iPSC from mouse blood and bone marrow

Peripheral blood (0.3–0.5 ml) was collected directly from the heart of mice at the time of necropsy, and bone and marrow cells were obtained from the femora and tibiae by flushing with medium. Blood samples were subjected to two rounds of erythrocyte lysis in ammonium chloride solution (STEMCELL Technologies, Vancouver, BC, Canada). A first lysis round with 10 ml for 15 min and at room temperature was followed by centrifugation (5 min at 219 × *g*) and a second lysis round with 3 ml for 15 min and at room temperature. Samples were then neutralized by adding 12 ml iPSC medium. Bone marrow samples were subjected to one round of erythrocyte lysis in ammonium chloride solution (3 ml for 5 min), followed by neutralization with 12 ml iPSC medium. Cells were pelleted, resuspended and plated on gelatin-coated plates with feeder layers. They were cultured in iPSC medium until iPSC colonies appeared. Cultures were routinely tested for mycoplasma and were always negative.

### Immunohistochemistry of tissue samples

Tissue samples were fixed in 10% neutral buffered formalin (4% formaldehyde in solution), paraffin-embedded and cut into 3 µm sections, which were mounted onto Superfrost® Plus slides (Thermo Fisher Scientific) and air-dried. Slides were deparaffinized in xylene and re-hydrated through a series of graded ethanol until water. Serial sections were stained with hematoxylin and eosin. For immunohistochemistry, an automated immunostaining platform was used (Ventana discovery XT; Roche, Madrid, Spain). Antigen retrieval was first performed with high pH buffer (CC1m, Roche). Endogenous peroxidase was blocked with 3% hydrogen peroxide and slides were then incubated with the appropriate primary antibodies. These were anti-Oct4 (1:500, ab19857, Abcam), anti-β-Galactosidase (1:3000, AM3A9A, CNIO Monoclonal Antibodies Core Unit), Tfe3 (1:500, HPA023881, Atlas Antibodies, Bromma, Sweden) or PCNA (1:1000, NA03, Merck). After the primary antibody, slides were incubated with the species-matched secondary antibodies conjugated with horseradish peroxidase (Chromomap, Ventana, Roche). Immunohistochemical reactions were developed using 3,3′-diaminobenzidine tetrahydrochloride (DAB, Ventana, Roche) as a chromogen, and nuclei were counterstained with hematoxylin. Finally, the slides were dehydrated, cleared and mounted with a permanent mounting medium (Tissue-Tek Glas Mounting Media, Sakura, Tokyo, Japan) for microscopic evaluation. Whole digital slides were acquired with a slide scanner (Mirax Scan, Zeiss, Jena, Germany), and images captured with the Pannoramic Viewer Software (3DHISTECH, Budapest, Hungary).

### Uncropped Images

Uncropped images of the gels displayed in the main figures are presented in Supplementary Fig. 13

### Data availability

All data generated or analyzed during this study are included in this published article (and its supplementary information files). Raw sequencing data are available via the accession code SRP144114 (https://www.ncbi.nlm.nih.gov/Traces/study/?acc=SRP144114). All additional data available upon reasonable request.

## Electronic supplementary material

Supplementary Information

Description of Additional Supplementary Files

Supplementary Data 1

Supplementary Data 2

Supplementary Movie 1

Supplementary Movie 2
