# Supplementary material for: AAV vector-mediated in vivo reprogramming into pluripotency
Source: Nat Commun. 2018 Jul 9;9:2651. doi: 10.1038/s41467-018-05059-x (PMC6037684; doi:10.1038/s41467-018-05059-x)
Supplement: Supplementary file 1 — Supplementary Information [file 41467_2018_5059_MOESM1_ESM.pdf]

Supplementary Information for

AAV vector-mediated *in vivo* reprogramming into pluripotency

Senís et al.

Supplementary Table 1. Scoring of the expression of pluripotency markers in 22 different *in vitro* generated iPSC clones.

	IVT-iPSC																					
Clone #	**1**	**2**	3	4	5	**7**	**8**	**9**	**10**	**12**	13	**14**	**19**	**20**	21	**22**	23	**24**	25	**26**	27	28
**AP**	**√**	**√**	√	√	√	**√**	**√**	**√**	**√**	**√**	√	**√**	**√**	**√**	√	**√**	√	**√**	√	**√**	√	√
**Oct-3/4 (IF)**	**√**	**√**	√	√	√	**√**	**√**	**√**	**√**	**√**	√	**√**	**√**	**√**		**√**		**√**	√	**√**	√	√
**Sox2 (IF)**	**√**	**√**	√	√	√	**√**	**√**	**√**	**√**	**√**	√	**√**	**√**	**√**		**√**		**√**	√	**√**	√	√
**Nanog (IF)**	**√**	**√**		√		**√**	**√**	**√**	**√**	**√**	√	**√**	**√**	**√**	√	**√**	√	**√**		**√**		√
**SSEA-1 (IF)**	**√**	**√**	√	√	√	**√**	**√**	**√**	**√**	**√**	√	**√**	**√**	**√**	√	**√**	√	**√**	√	**√**		√
**mNanog (RT)**	**√**	**√**				**√**	**√**	**√**	**√**	**√**		**√**	**√**	**√**	√	**√**		**√**		**√**		√
**mOct-3/4 (RT)**	**√**	**√**	√	√	√	**√**	**√**	**√**	**√**	**√**	√	**√**	**√**	**√**	√	**√**	√	**√**		**√**	√	√
**mEsg1 (RT)**	**√**	**√**	√	√	√	**√**	**√**	**√**	**√**	**√**	√	**√**	**√**	**√**	√	**√**	√	**√**	√	**√**	√	√
**mRex1 (RT)**	**√**	**√**		√	√	**√**	**√**	**√**	**√**	**√**	√	**√**	**√**	**√**	√	**√**	√	**√**	√	**√**	√	√
**mGdf3 (RT)**	**√**	**√**	√	√	√	**√**	**√**	**√**	**√**	**√**		**√**	**√**	**√**	√	**√**	√	**√**	√	**√**	√	√
**mERas (RT)**	**√**	**√**	√	√	√	**√**	**√**	**√**	**√**	**√**	√	**√**	**√**	**√**	√	**√**		**√**		**√**	√	√
**mEcat1 (RT)**	**√**	**√**	√	√		**√**	**√**	**√**	**√**	**√**		**√**	**√**	**√**	√	**√**		**√**		**√**		

AP, alkaline phosphatase; IF, immunofluorescence; RT, RT-PCR; √, expression; bold/underlined, clones that express the full set of pluripotency markers.

Note that only clones are listed that were tested in both assays, IF and RT. Hence, clones #11, 15 and 16 were omitted from the table since they were not studied by IF (see Supplementary Fig. 5b for RT data).

Supplementary Table 2. Histopathological organ analysis of mice (three per dose) sacrificed two weeks post-injection.

Organ	Dose (vg/mouse) of AAV8-SFFV-hCO-OKSM vectors	
	**5×10^{10}**	**2×10^{11}**
Liver	Mild increase in nucleus-to-cytoplasm (N/C) ratio<br>Foci of undifferentiated cells	Moderate increase in N/C ratio<br>Moderate pleomorphism<br>Increased mitoses<br>Polynucleated hepatocytes<br>Foci of undifferentiated cells
Pancreas	Mild, multifocal increase in N/C ratio	
Intestine	Basal cells active: hyperchromatic vesicular nuclei<br>Increased mitoses	
Heart	Larger and pleomorphic nuclei with stippled chromatin and prominent nucleoli<br>Scattered degenerated fibers<br>Regenerating fibers with 3-4 nuclei in a row<br>Arteries: nuclear pleomorphism and disorganized tunica media	
Muscle	Low numbers of basophilic polynucleated fibers (1/3 mice)	Multifocal fiber hyperplasia (2/3)
Bone marrow	No pathological findings	Slight increase in mature granulocytes
Spleen	Reactive: mild, diffuse hyperplasia of germinal centers<br>Extramedullary hematopoiesis (EMH)	
Thymus	No pathological findings	
Kidney	No pathological findings	
Lung	No pathological findings	
Stomach	No pathological findings	
Adipose tissue	No pathological findings	
Lymph nodes	Reactive<br>Mildly subcapsular increase in mast cells	
Brain	Mild ventricle dilation<br>Mild nuclear activation of the choroid plexus	

Supplementary Table 3. Histopathological organ analysis of mice (three per dose) sacrificed four weeks post-injection.

Organ	Dose (vg/mouse) of AAV8-SFFV-hCO-OKSM vectors	
	5×10^{10}	2×10^{11}
Liver	Moderate increase in N/C ratio<br>Moderate pleomorphism<br>Increased mitoses<br>Polynucleated hepatocytes	Moderate increase in N/C ratio<br>Moderate pleomorphism<br>Increased mitoses<br>Polynucleated hepatocytes<br>Foci of undifferentiated cells<br>Teratomas (2/3)
Pancreas	Mild, multifocal increase in N/C ratio	
Intestine	Basal cells active: hyperchromatic vesicular nuclei<br>Increased mitoses	
Heart	Larger and pleomorphic nuclei with stippled chromatin and prominent nucleoli<br>Scattered degenerated fibers<br>Regenerating fibers with 3-4 nuclei in a row<br>Arteries: nuclear pleomorphism and disorganized tunica media	
Muscle	Fiber regeneration: nuclei in rows, increased N/C ratio, mild pleomorphism, stippled chromatin<br>Arteries: mild smooth muscle cell pleomorphism and scattered mast cell infiltration	
Bone marrow	Mild aplasia<br>Mild hemosiderosis	
Spleen	Reactive: moderate, diffuse follicle hyperplasia	
Thymus	Cyst with respiratory epithelia (1/3)	No pathological findings
Kidney	Mild, multifocal chronic interstitial nephritis	Mild, multifocal pyelitis and tubulonephrosis
Lung	No pathological findings	
Stomach	No pathological findings	Mild pleomorphism and dilated glands (1/3)
Adipose tissue	No pathological findings	
Lymph nodes	Reactive<br>Mildly subcapsular increased in mast cells	
Brain	Mild ventricle dilation<br>Mild nuclear activation of the choroid plexus	

Supplementary Table 4. Histopathological organ analysis of mice (three per dose) sacrificed more than four weeks post-injection.

Organ	Dose (vg/mouse) of AAV8-SFFV-hCO-OKSM vectors	
	5×10^{10}	2×10^{11}
Liver	Moderate increase in N/C ratio<br>Moderate pleomorphism<br>Increased mitoses<br>Polynucleated hepatocytes	Moderate increase in N/C ratio<br>Moderate pleomorphism<br>Increased mitoses<br>Polynucleated hepatocytes<br>Multifocal to coalescent foci of undifferentiated cells<br>Teratomas (3/3)
Pancreas	Mild, multifocal increase in N/C ratio<br>Teratomas (1/3)	Mild, multifocal increase in N/C ratio<br>Foci of undifferentiated cells in peritoneum (metastasis from liver)
Intestine	Basal cells active: hyperchromatic vesicular nuclei<br>Increased mitoses	Basal cells active: hyperchromatic vesicular nuclei<br>Increased mitoses<br>Slightly undifferentiated crypts
Heart	Larger and pleomorphic nuclei with stippled chromatin and prominent nucleoli<br>Scattered degenerated fibers<br>Regenerating fibers with 3-4 nuclei in a row<br>Increased numbers of binucleated cardiomyocytes<br>Arteries: nuclear pleomorphism and disorganized tunica media	
Muscle	Fiber regeneration<br>Arteries: mild smooth muscle cell pleomorphism and increased mast cell infiltration	Mild fiber regeneration<br>Increased number of cells<br>Increased mast cell infiltration (perivascular and perineural)
Bone marrow	Slight increase in granulocytes (1/3)	Mild aplasia<br>Mild hemosiderosis
Spleen	Reactive: moderate, diffuse follicle hyperplasia<br>Severe EMH (1/3)	Mild, multifocal germinal center hyperplasia
Thymus	Metastatic lymphoma (1/3)	Teratoma (1/3)
Kidney	Mild, multifocal pyelitis and tubulonephrosis	Mild, multifocal chronic interstitial nephritis<br>Moderate tubulonephrosis<br>Multifocal areas of tubular regeneration
Lung	No pathological findings	Foci of intravascular and parenchymal undifferentiated cells (metastasis from liver) (1/3)
Stomach	Mild pleomorphism and dilated glands (2/3)	Mild pleomorphism and dilated glands (3/3)

Adipose tissue	No pathological findings	
Lymph nodes	Lymphoma (primary site) (1/3)	Reactive
Brain	Mild ventricle dilation<br>Mild nuclear activation of the choroid plexus	

Supplementary Table 5. Summary of *in vivo* reprogramming experiments where teratomas became palpable.

Assay	Mouse genotype	Dose (vg/vector)	AAV vectors	Mice with ≥1 teratoma	Mouse #	Hepatic teratomas	Extra-hepatic teratomas or metastasis	Other tumors	Latency (weeks)	Other results
#1	wild-type	5×10^{10}	OKSM	33% (n=3)	1	---	1 (P)	---	38	
					2	---	---	1 (LN)	---	
					3	---	---	---	---	
#2	wild-type	2×10^{11}	OKSM	100% (n=3)	1	2*	---	---	7	* iPSC (Bl, BM, Te)
					2	4*	3 (L, F, T)	---	8	* iPSC (Te)
					3	2*	---	---	10	* iPSC (Te)
#3	wild-type	2×10^{11}	OKSM	100% (n=4)	1	3	5 (BM, F, L, LN, K)	---	7	
					2	2	2 (BM, L)	---	7	
					3	>7	1 (L)	---	8	
					4	3	7 (F, I, K, L, LN, P, Thl)	---	13	
#4	Rosa26:: LSL-LacZ	2×10^{11}	OKSM + TTR-Cre	100% (n=3)	1	>7	5 (F, L, LN, P, S)	---	7	Hepatocytes: 100% β-galactosidase + teratomas: 0-100%
					2	7	2 (F, L)	---	7	
					3	>7	5 (F, K, L, LN, P)	---	8	
#5	wild-type	1×10^{12}	OKSM	100% (n=3)	1	>7	3 (F, L, LN)	---	6	
					2	>7	3 (F, K, L)	---	6	
					3	>7	3 (F, L, LN)	---	6	
#6	wild-type	1×10^{12}	OKS + SFFV-GFP	33% (n=3)	1	---	1 (P)*	---	53	* iPSC (Te) *In vivo* reprogramming without c-Myc
					2	---	---	---	---	
					3	---	---	---	---	

Bl, blood; BM, bone marrow; F, fat; I, intestine; K, kidney; L, lung; LN, lymph node; P, pancreas; S, stomach; T, thymus; Te, teratoma; Th, thoracic cavity; vg, vector genomes. * indicates the origin of the iPSC

Supplementary Table 6. Primers used in this study.

Name	Sequence (5'-3')
BGHpoly(A)_Rev	CTCCCCAGCATGCCTGCTATTG
CMV_5'	TGCCCAGTACATGACCTTATGG
CMV_3'	GAAATCCCCGTGAGTCAAACC
CMV_hOct4_For_BamHI	GCCGGTGGATCCACCGG
hCO c-myc_For_BamHI	cgGGATCCACCGGTGCCACCATGCCCCTCAACGTTAGCTTCACC
hCO c-myc_Rev_SalI	gttgcgGTCGACTCATCACGCACAAGAGTTCC
hKlf4_For_BamHI	cgGGATCCACCGGTGCCACCATGGCTGTGTCCGACGCCC
hKlf4_Rev_SalI	gttgcgGTCGACTCAGAAGTGTCTCTTCATGTGCAGAGCCAG
hOct4_For_BamHI	GCCGGTGGATCCACCGG
hOct4_Rev_SalI	gttgcgGTCGACTCAGTTGCTATGCATGGGGCTGCC
hSox2_for_BamHI	cgGGATCCACCGGTGCCACCATGTACAACATGATGGAAACCGAGCTGAA
hSox2_rev_SalI	gttgcgGTCGACTCACATGTGGCTCAGGGGCAGG
Klf4_For_NotI	aaatatGCGGCCGCATGGCTGTCAGCGACGCTCTG
LCI	GACCCGGGAGATCTGAATTC
LCII	GATCTGAATTCAGTGGCACAG
LC1nrLAM1800-1822	[Phos]-TAG-BC-CACCTAACTGCTGTGCCACTGAATTCAGATCTCCCGGGT-[DDC]
mActin_F	GGCACCACACCTTCTACAATG
mActin_R	GTGGTGGTGAAGCTGTAGCC
MegaLinker primer	AATGATACGGCGACCACCGAGATCTACACTCTTTCCCTACACGACGCTCTTCCGATCT-BC-CAGGCATGCTGGGGAGAGATC
mGAPDH_For	TTGATGGCAACAATCTCCAC
mGAPDH_Rev	CGTCCCGTAGACAAAATGGT
mHprt_For	GCTGGTGAAAAGGACCTCT
mHprt_Rev	CCAACAACAAACTTGTCTGG
MiSAAV180	AATGATACGGCGACCACCGAGATCTACACTCTTTCCCTACACGACGCTCTTCCGATCTCACTCTAGCTCAGGCATGCTGGGGAGAGATC
MiSAAV181	AATGATACGGCGACCACCGAGATCTACACTCTTTCCCTACACGACGCTCTTCCGATCTTATACGACGTCAGGCATGCTGGGGAGAGATC
MiSAAV182	AATGATACGGCGACCACCGAGATCTACACTCTTTCCCTACACGACGCTCTTCCGATCTCGTATCTACACAGGCATGCTGGGGAGAGATC
MiSAAV183	AATGATACGGCGACCACCGAGATCTACACTCTTTCCCTACACGACGCTCTTCCGATCTAGCTAGACGCCAGGCATGCTGGGGAGAGATC
MiSAAV184	AATGATACGGCGACCACCGAGATCTACACTCTTTCCCTACACGACGCTCTTCCGATCTATCACTACTGCAGGCATGCTGGGGAGAGATC
MiSAAV185	AATGATACGGCGACCACCGAGATCTACACTCTTTCCCTACACGACGCTCTTCCGATCTAGATACTCACCAGGCATGCTGGGGAGAGATC
MiSAAV186	AATGATACGGCGACCACCGAGATCTACACTCTTTCCCTACACGACGCTCTTCCGATCTCGCGTGCATGCAGGCATGCTGGGGAGAGATC
MiSAAV187	AATGATACGGCGACCACCGAGATCTACACTCTTTCCCTACACGACGCTCTTCCGATCTCTGCAGACTCCAGGCATGCTGGGGAGAGATC
MiSAAV188	AATGATACGGCGACCACCGAGATCTACACTCTTTCCCTACACGACGCTCTTCCGATCTCGTAGCAGACCAGGCATGCTGGGGAGAGATC
MiSAAV189	AATGATACGGCGACCACCGAGATCTACACTCTTTCCCTACACGACGCTCTTCCGATCTAGTCAGTACGCAGGCATGCTGGGGAGAGATC
MiSAAV190	AATGATACGGCGACCACCGAGATCTACACTCTTTCCCTACACGACGCTCTTCCGATCTCTACTGTCGCCAGGCATGCTGGGGAGAGATC

MiSAAV191	AATGATACGGCGACCACCGAGATCTACACTCTTTCCCTACACGACGC TCTTCCGATCTACTGAGTCATCAGGCATGCTGGGGAGAGATC
MiSAAV192	AATGATACGGCGACCACCGAGATCTACACTCTTTCCCTACACGACGC TCTTCCGATCTCTCATGAGAGCAGGCATGCTGGGGAGAGATC
MiSAAV193	AATGATACGGCGACCACCGAGATCTACACTCTTTCCCTACACGACGC TCTTCCGATCTTATCGTGAGACAGGCATGCTGGGGAGAGATC
MiSAAV194	AATGATACGGCGACCACCGAGATCTACACTCTTTCCCTACACGACGC TCTTCCGATCTCATGTACTCACAGGCATGCTGGGGAGAGATC
MiSAAV195	AATGATACGGCGACCACCGAGATCTACACTCTTTCCCTACACGACGC TCTTCCGATCTATAGTAGAGTCAGGCATGCTGGGGAGAGATC
MiSAAV196	AATGATACGGCGACCACCGAGATCTACACTCTTTCCCTACACGACGC TCTTCCGATCTACAGTGTCGACAGGCATGCTGGGGAGAGATC
MiSAAV197	AATGATACGGCGACCACCGAGATCTACACTCTTTCCCTACACGACGC TCTTCCGATCTACGAGTCGTCCAGGCATGCTGGGGAGAGATC
MiSAAV198	AATGATACGGCGACCACCGAGATCTACACTCTTTCCCTACACGACGC TCTTCCGATCTCACGACATAGCAGGCATGCTGGGGAGAGATC
MiSAAV199	AATGATACGGCGACCACCGAGATCTACACTCTTTCCCTACACGACGC TCTTCCGATCTCGCATCTGTACAGGCATGCTGGGGAGAGATC
mScarlet_fw	ttttACCGGTGCCACCATGGTGAGCAAGG
mScarlet_re	aaaaGCTAGCTTATCTAGATCCGGTGGATCCCG
mTurquoise_fw	ttttACCGGTCGCCACCATGGTG
mTurquoise_re	aaaaGCTAGCTTATCTAGATCCGGTGGATCCCggg
Oct4_Klf4_For	cagcctGCGGCCGCCATGGC
Oct4_Klf4_For_HindIII	cccAAGCTTATGGCTGGACACCTGGCTTCAGA
Oct4_Klf4_Rev_SalI	gttgcgGTCGACTTAAAAGTGCCTCTTCATGTGTAAGGCAAGGT
Oct4_Rev_SalI	gttgcgGTCGACTTAGTTTGAATGCATGGGAGAGCCC
P1 (nrLAM-PCR)	[Biotin]-CATCGCATTGTCTGAGTAGGTG
P2 (nrLAM-PCR)	[Biotin]-GAGGAAATTGCATCGCATTGTC
P3 (nrLAM-PCR)	[Biotin]-GACAGTGGGAGTGGCACCTTCC
P4 (nrLAM-PCR)	GAAGACAATAGCAGGCATGCTGG
P5 (nrLAM-PCR)	[Biotin]-GTAGGTGTCATTCTATTCTGGG
Pseq (nrLAM-PCR)	CAGGCATGCTGGGGAGAGATC
qRTPCR_hOct4_For	CAGCATCGAGAACAGAGTGC
qRTPCR_hOct4_Rev	ACACTCTCACGACGTCCTTTTCC
qRTPCR_hKlf4_For	GCAGAGAGAAAACACTGCGG
qRTPCR_hKlf4_Rev	AAGGTCTGCCGGGCAGCAC
qRTPCR_hSox2_For	AAAGAGCACCCCGACTACAAG
qRTPCR_hSox2_Rev	CTCCGCTGGCCATAGAATTGC
qRTPCR_hcmyc_For	TTCTCCCTTCGGGGAGACAAC
qRTPCR_hcmyc_Rev	CCATGTCTCCTCCCAGCAG
qRTPCR_mGapdh_For	AGGTCGGTGTGAACGGATTTG
qRTPCR_mGapdh_Rev	TGTAGACCATGTAGTTGAGGTCA
qRTPCR_mOct4_For	TAGGTGAGCCGTCTTTCCAC
qRTPCR_mOct4_Rev	GCTTAGCCAGGTTCGAGGAT
qRTPCR_mSox2_For	GAAACGACAGCTGCGGAAA
qRTPCR_mSox2_Rev	TCTAGTCGGCATCACGGTTTT
qRTPCR_mNanog_For	TTGCTTACAAGGGTCTGCTACT
qRTPCR_mNanog_Rev	ACTGGTAGAAGAATCAGGGCT
RSV_R	CCCGGGTTGTGGCAAGCTTGGAGGTGCACACCAATG
SFFV_5'	AAGAACAGATGGTCCCCAG
SFFV_3'	GAACAGAAGCGAGAAGCAG

SFFV_For_EcoRI	cgGAATTCAGCTAGCTGCAGTAACGCCATTTTGC
Sox2_cmyc_For_NotI	aaatatGCGGCCGCATGTATAACATGATGGAGACGGAGC
Sox2_cmyc_For_XhoI	ccgCTCGAGATGTATAACATGATGGAGACGGAGC
Sox2_cmyc_Rev_SalI	ttttgacGTCGACTTATGCACCAGAGTTTCGAAGCT
Sox2_cmyc_Rev_NotI	aaatatGCGGCCGCTTATGCACCAGAGTTTCGAAGCT
Sox2_Rev_SalI	gttgcgGTCGACTTACATGTGCGACAGGGGC
TTR_5'	TGTTCCGATACTCTAATCTCCC
TTR_3'	TATACCCCCTCCTTCCAACC

Upper case + underlined, restriction sites; lower case, overhangs or stuffer sequences; [Biotin], biotinylated at the 5' end; [Phos], phosphorylated at the 5’ end (needed for ligation); -BC-, 10 nt long barcode used for sample recognition in a pooled sequencing library; [DDC], dideoxycytidine at 3' end to prevent self-ligation

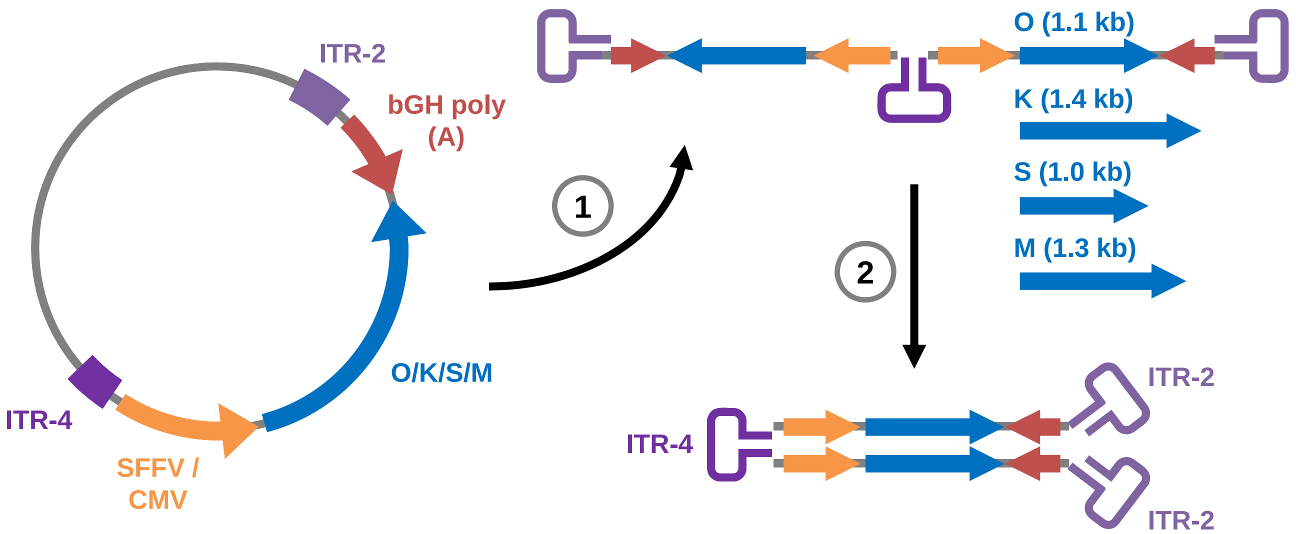

Supplementary Figure 1. Cloning and packaging of self-complementary (sc)AAV vector genomes encoding human codon-optimized (hCO) cDNAs of Oct-3/4 (O), Klf4 (K), Sox2 (S) or c-Myc (M) (one cDNA per vector). Shown on the left are the AAV vector plasmids in which the cDNAs are under the control of the CMV or SFFV promoter, terminated by a bovine growth hormone (bGH) polyadenylation signal and flanked by inverted terminal repeats (ITR) of AAV serotypes 2 or 4. The AAV4 ITR carries a mutation that impairs ITR nicking during replication which is normally required for resolution of the ITR. Thus, replication continues through the mutated ITR, producing a single-stranded AAV genome carrying two inverted copies of the expression cassette that are separated by the mutated ITR and flanked by two wild-type ITRs (1). After uncoating, the viral DNA immediately folds back onto itself using the mutated ITR as a hinge and forming a double-stranded molecule (2) that rapidly and potently expresses the encoded O/K/S/M transgenes.

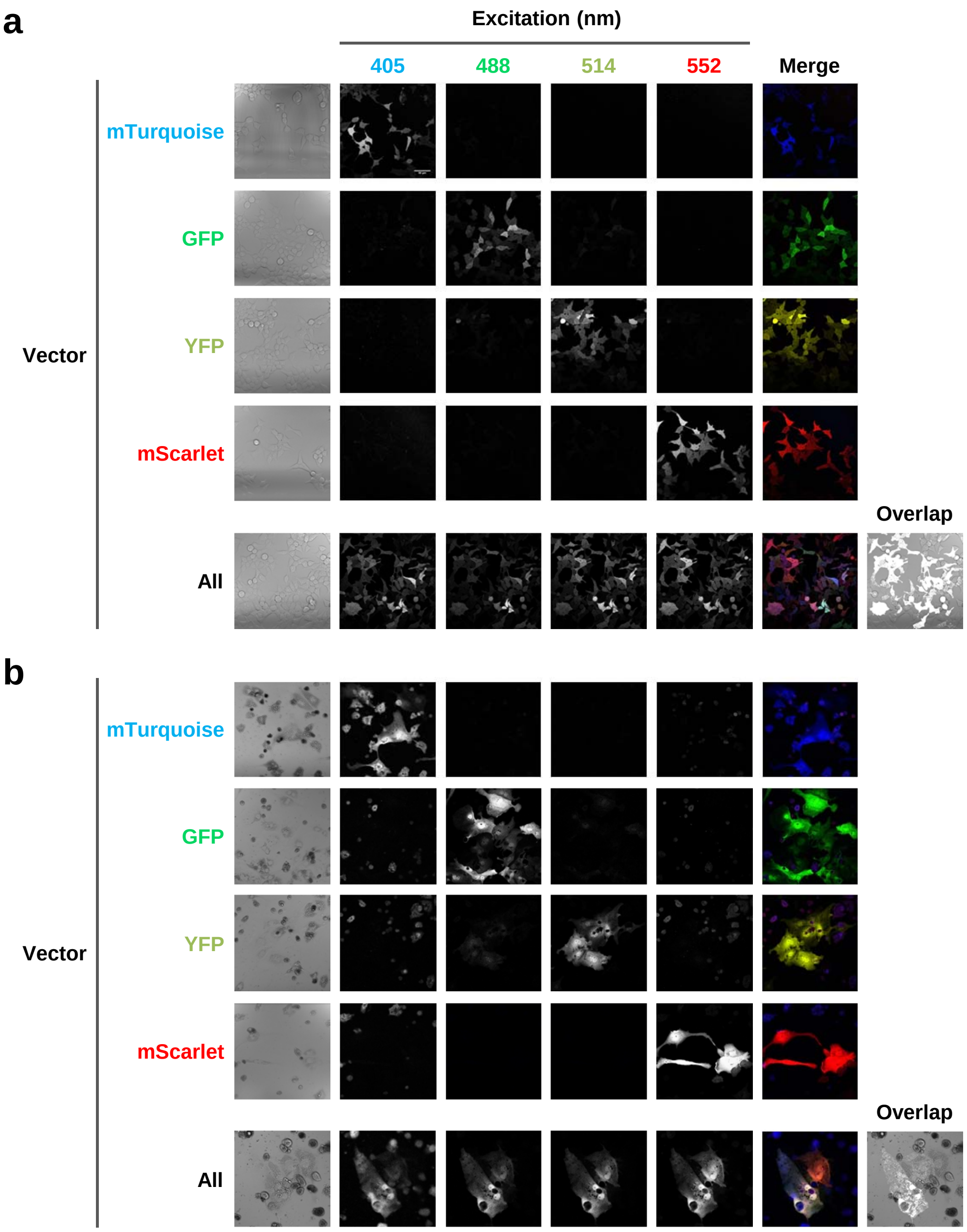

Supplementary Figure 2. Efficient co-expression of four transgenes delivered by four different AAV vectors. HEK293T cells (**a**) or primary murine hepatocytes (**b**) were (co-)transduced with four AAV vectors (multiplicity of infection of 10^4), each encoding one of the shown four fluorescent proteins. Cells labeled “All” received all four vectors simultaneously. Fluorescence was recorded on a confocal microscope using lasers with the indicated wavelengths for excitation. Column “Merge” shows an overlay of the pseudocolored signals measured at each setting. In panels “Overlap”, white pixels indicate co-expression of all four fluorescent proteins within the shown cells.

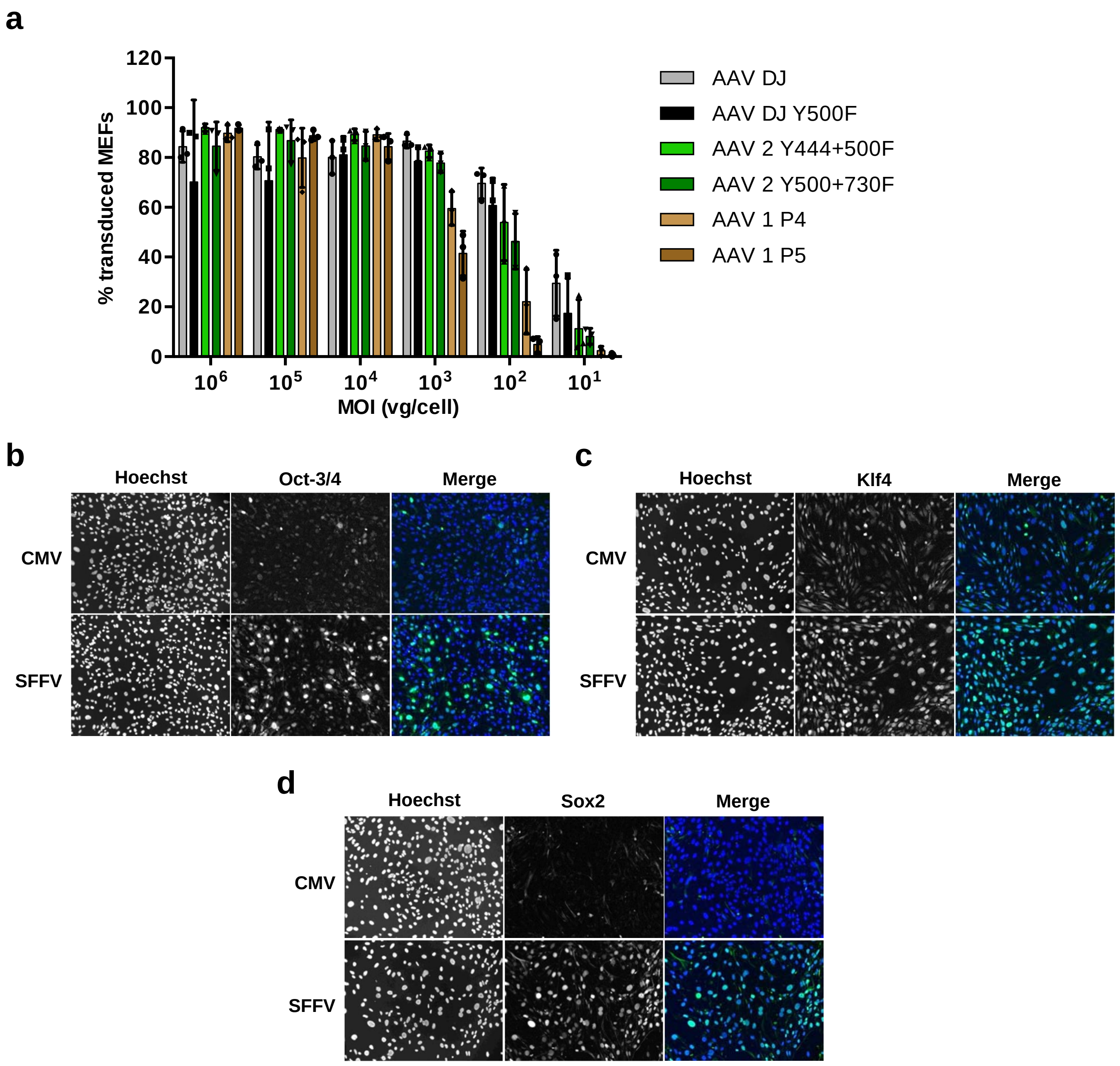

Supplementary Figure 3. Identification of a potent AAV capsid in MEF (see Methods for details on capsids). (**a**) MEF were transduced with purified AAV vectors encoding a YFP reporter at the indicated MOIs. Forty-eight hours latter, percentages of fluorescent cells were determined by flow cytometry. Error bars are S.D. (n = 3). (**b-d**) Comparison of hCO-O/KS expression in MEF from a CMV or SFFV promoter. Cells were transduced with the indicated AAV-DJ vectors at day 0 and 2 at a MOI of 4×10^4 vg per cell. At day 4, expression of Oct-374 (**b**), Klf4 (**c**) or Sox (**d**) was detected via immunofluorescence. All images were processed with ImageJ. Background was subtracted using the sliding paraboloid option.

a

	AAV-DJ-CMV-hCO-O:K:S:M stoichiometry					
MOI	1:1:1:1	2:1:1:1	4:1:1:1	6:1:1:1	8:1:1:1	10:1:1:1
1×10^5	1	1	1	4	1	1
1×10^4	3	2	12	1	2	3
1×10^3	5	5	10	5	9	0

b

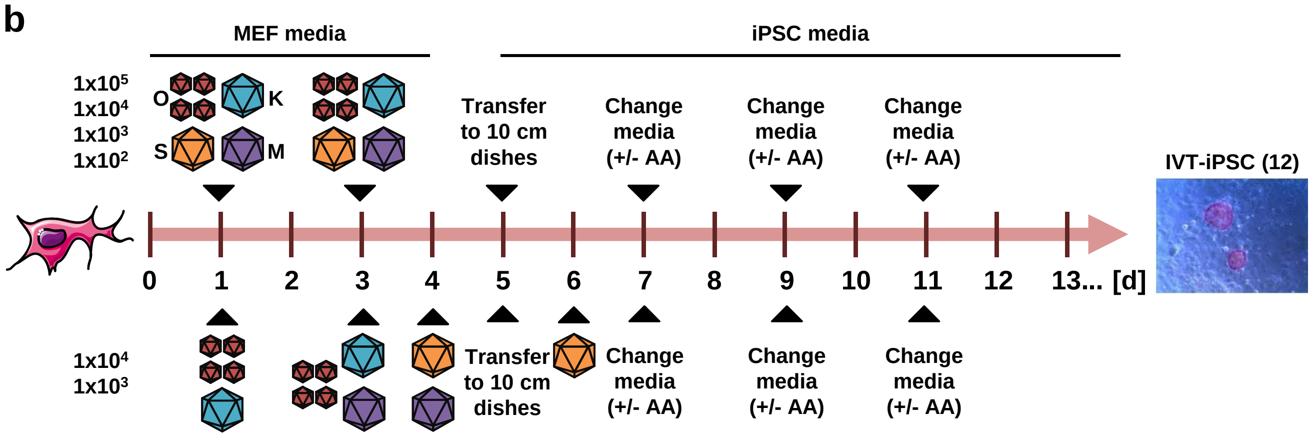

Promoter	MOI	Other	First iPSC colony observed at day	Reprogramming efficiency (max)	Number of experiments (n)
SFFV	$1x10^5$		-	0%	1
CMV	$1x10^4$		-	0%	1
SFFV	$1x10^4$		13	0.2%	4
SFFV	$1x10^4$	+ ascorbic acid (AA)	13*	0.2%	2
SFFV	$1x10^4$	sequential	23	0.1%	2
SFFV	$1x10^3$		13	0.01%	3
SFFV	$1x10^3$	+ ascorbic acid (AA)	13*	0.1%	2
SFFV	$1x10^3$	sequential	15	0.001%	2
SFFV	$1x10^2$		-	0%	1

Supplementary Figure 4. Optimization of the *in vitro* reprogramming protocol. (**a**) MEF were transduced at day 1 and 3 with AAV-DJ-CMV-hCO-OKSM at three different MOIs and using increasing levels of Oct-3/4 (1x, 2x, 4x, 6x, 8x or 10x more than the other three factors). From day 15 to 22, the cells were stained with alkaline phosphatase (AP) to detect reprogramming events. AP-positive areas appeared (their numbers are shown in the table), but none of them displayed proper iPSC colony morphology. (**b**) Additional reprogramming conditions tested. In the experiment represented above the arrow, MEF were transduced at days 1 and 3 with AAV-DJ-SFFV-hCO-OKSM with an O:K:S:M stoichiometry of 4:1:1:1, using the different MOIs indicated. The experiment below the arrow represents a sequential transduction protocol. MEF were transduced with AAV-DJ-SFFV-hCO-OKSM with an O:K:S:M stoichiometry of 4:1:1:1, using the two different MOIs indicated and the following regime: OK at day 1 in the evening; OKM at day 3 in the morning; MS at day 4 in the evening; S at day 6 in the morning. The table summarizes all results, sorted by decreasing AAV vector MOIs. * indicates that more iPSC colonies were observed at this early time point than under the same conditions without ascorbic acid (AA). This figure contains an element from Servier Medical Art (https://smart.servier.com).

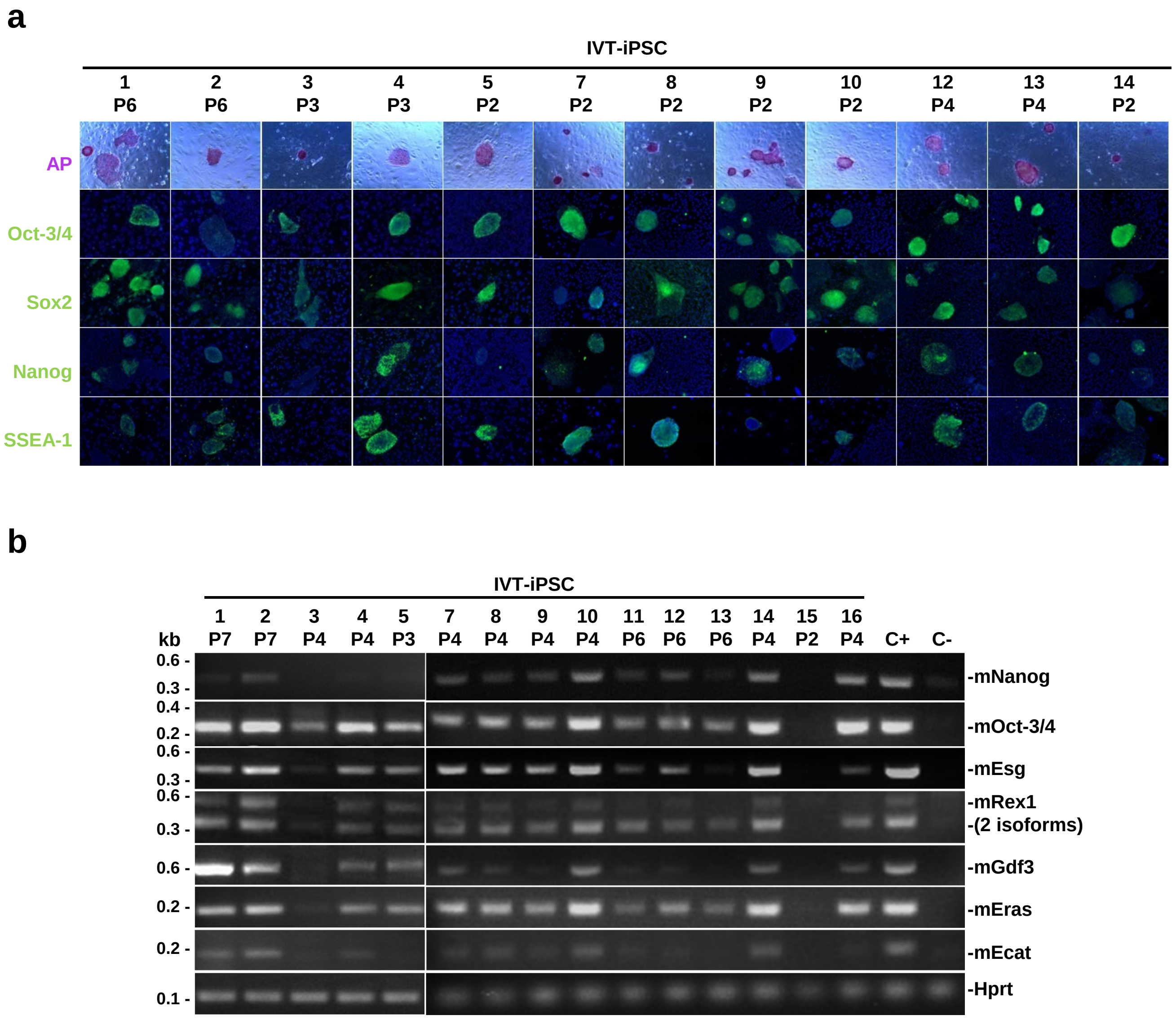

Supplementary Figure 5. Pluripotency marker expression in iPSC reprogrammed *in vitro* with AAV-DJ-SFFV-hCO-OKSM. (**a**) Alkaline phosphatase (AP) staining and immunofluorescence (IF) to detect the expression of Oct-3/4, Sox2, Nanog and SSEA-1. The secondary antibody was coupled with Alexa Fluor 488. The images were processed with ImageJ. Shown are Hoechst and Alexa Fluor 488 merges. P, passage number of each clone at which the images were taken. (**b**) Reverse transcription-PCR (RT-PCR) to detect expression of the depicted pluripotency markers. Hprt was used as housekeeper. P, passage number; C+, iPSC generated with a lentiviral vector encoding human codon-optimized OKSM (positive control); C-, MEF (negative control).

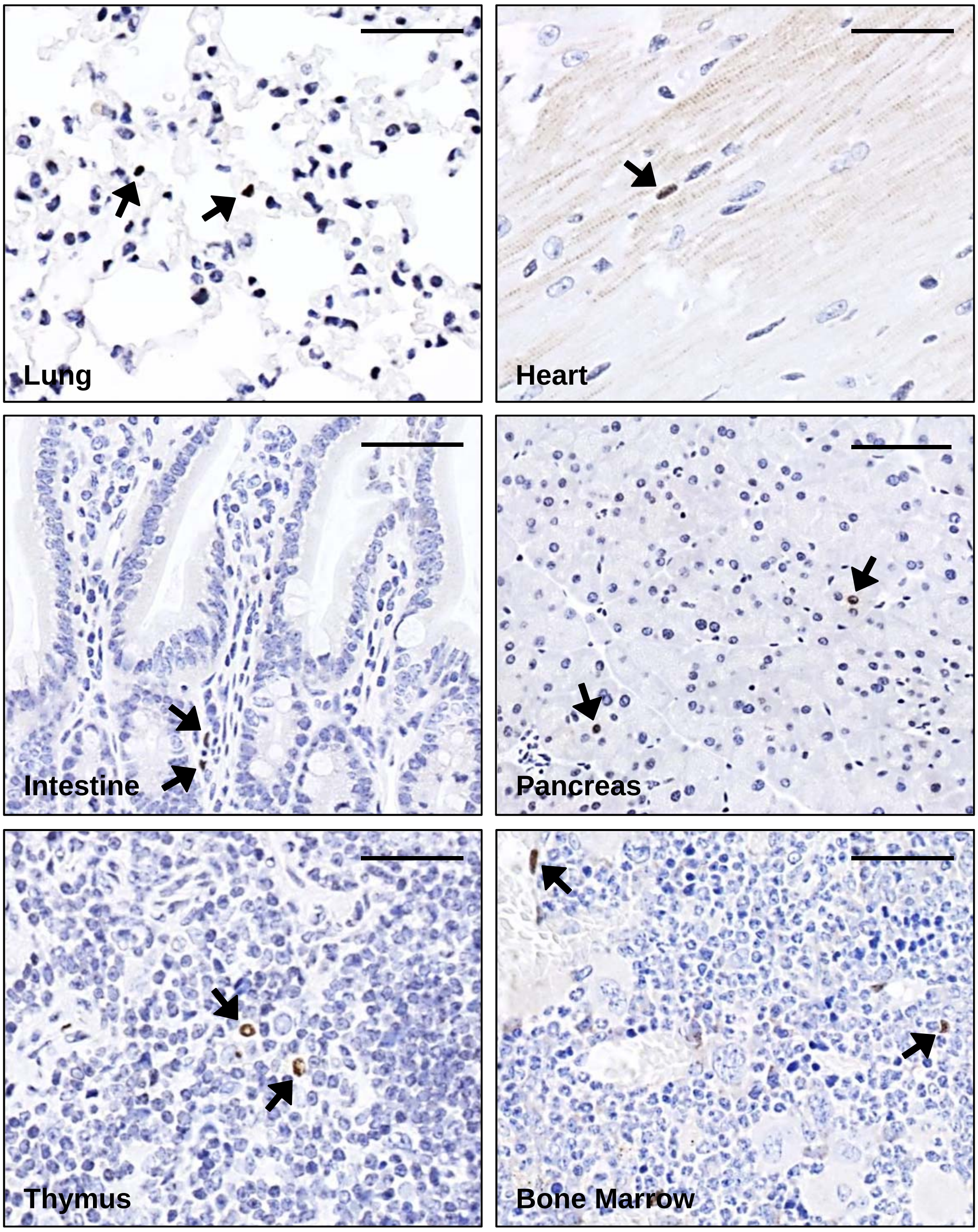

Supplementary Figure 6. Immunohistochemical detection of Oct-3/4 expression in various organs of mice injected with $5x10^{10}$ viral genomes per vector (scAAV8 SFFV-hCO-O/K/S/M; Oct-3/4 data are shown as a representative example). Tissues were extracted two weeks after vector injection (n = 3). Nuclei were counterstained with hematoxylin. Arrows highlight examples of Oct-3/4-positive cells. Scale bars = 100 μm (for lung, heart, pancreas, thymus and bone marrow) or 50 μm (for intestine).

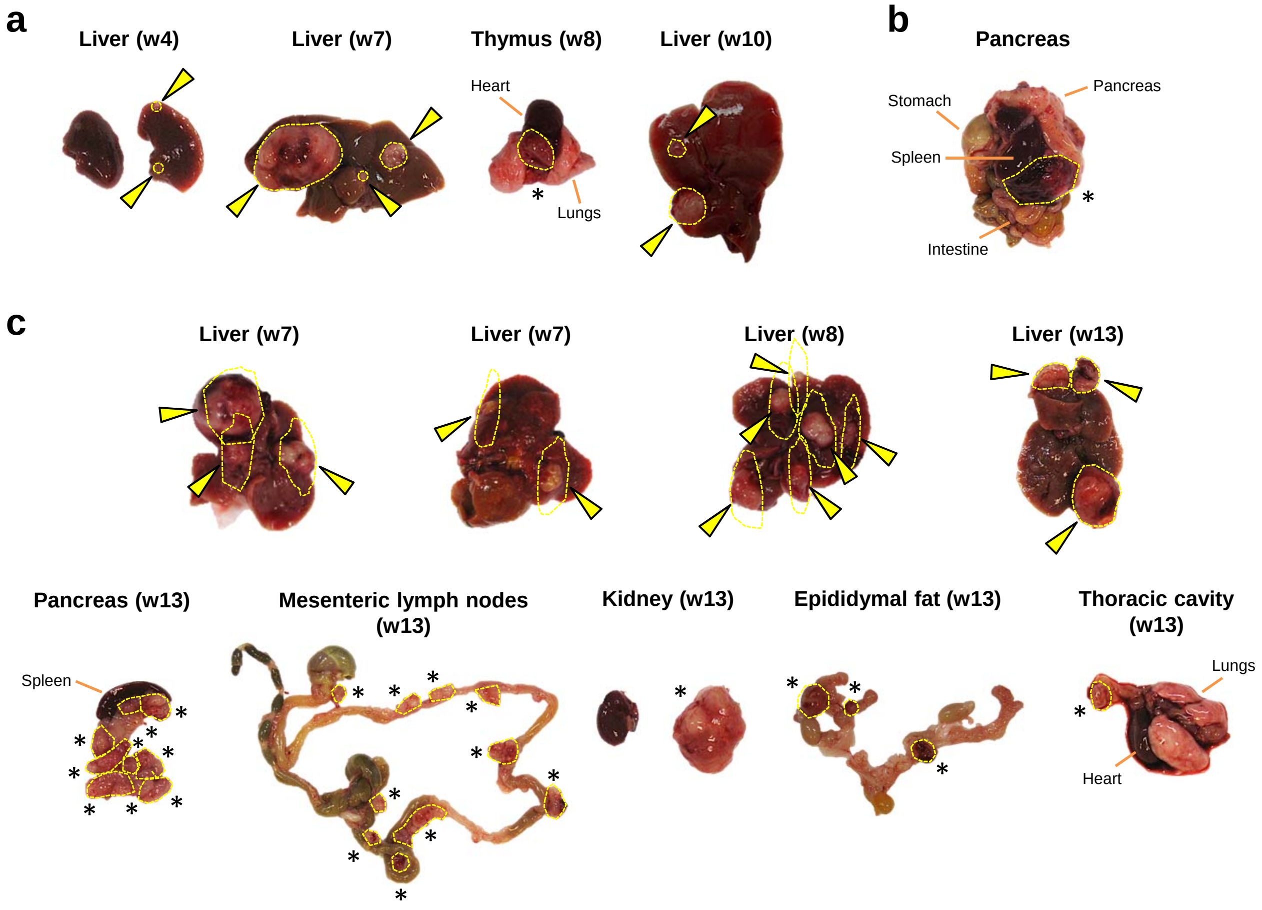

Supplementary Figure 7. Results of *in vivo* reprogramming experiments using scAAV8 SFFV-hCO-O/K/S/M vectors. (**a**) Images of livers and a thymus with teratomas extracted at the indicated week (w) post-injection from mice treated with 2×10^{11} vg per vector and per mouse (assay #2, Supplementary Table 5). (**b**) Images of the organs from the abdominal cavity with teratomas extracted at month 9 post-injection from a mouse treated with 5×10^{10} vg per vector (assay #1, Supplementary Table 5). (**c**) Images of organs with teratomas extracted at the indicated week post-injection from mice treated with 2×10^{11} vg per vector and per mouse (assay #3, Supplementary Table 5). Yellow arrows (livers) and stars (all other organs) indicate teratomas.

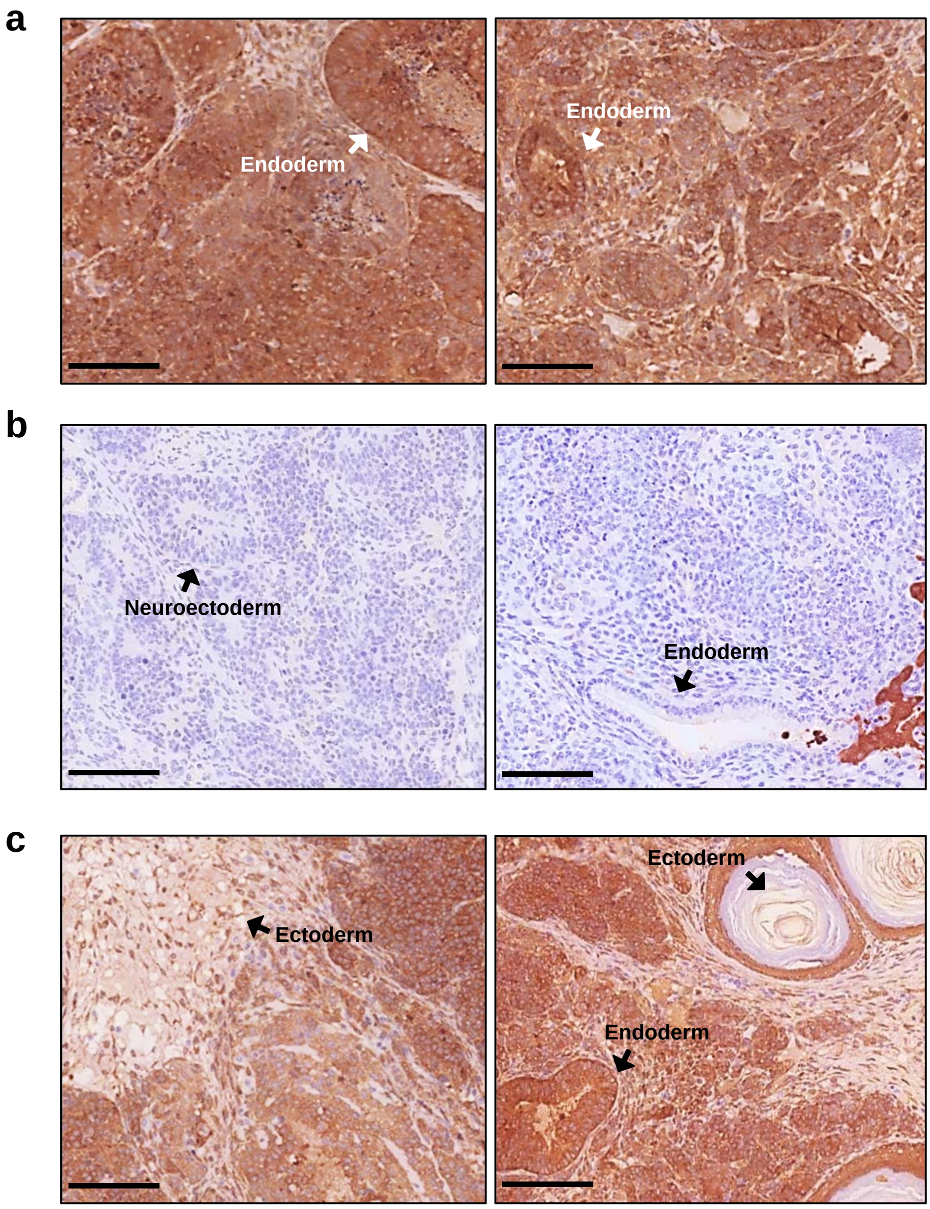

Supplementary Figure 8. β-galactosidase stainings of teratoma sections in LSL-LacZ mice injected with 2×10^{11} vg of OSKM- and Cre-encoding scAAV8 (n = 3)**,** and sacrificed seven or eight weeks post-injection. (**a**) Examples of teratomas that were almost 100% positive for β-galactosidase. (**b**) Examples of teratomas that were almost 100% negative for β-galactosidase. (**c**) Examples of teratomas with mosaic expression of β-galactosidase. Scale bars = 100 µm.

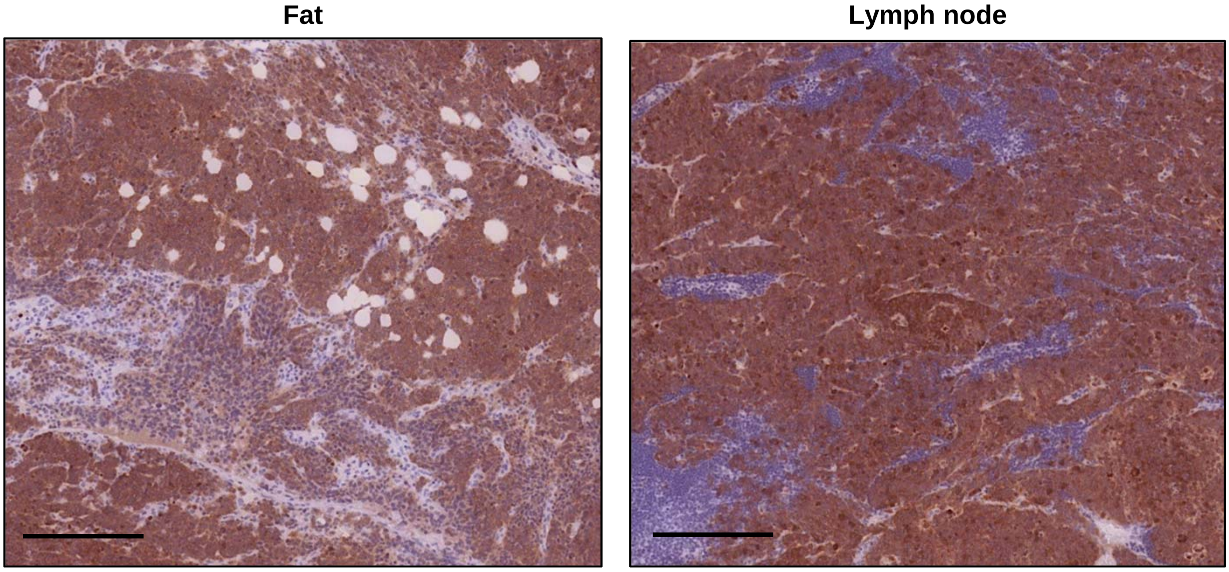

Supplementary Figure 9. β-galactosidase stainings of masses of undifferentiated cells found in extra-hepatic locations (in this example, fat and mesenteric lymph nodes) in a LSL-LacZ mouse injected with 2×10^{11} vg of OSKM- and Cre-encoding scAAV8 and sacrificed eight weeks post-injection. Scale bars = 200 µm.

a

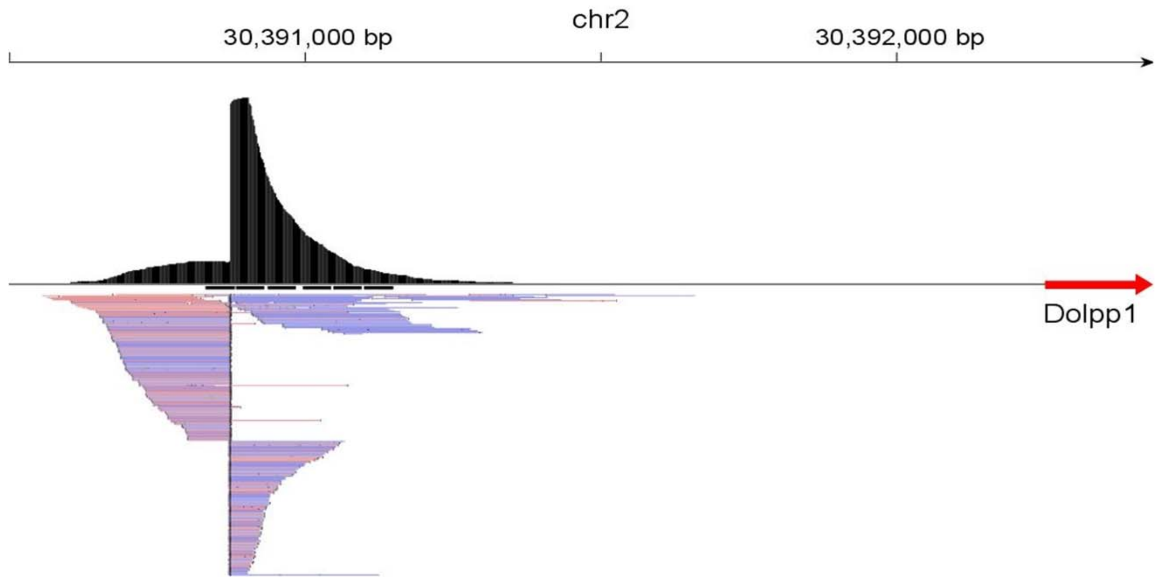

b

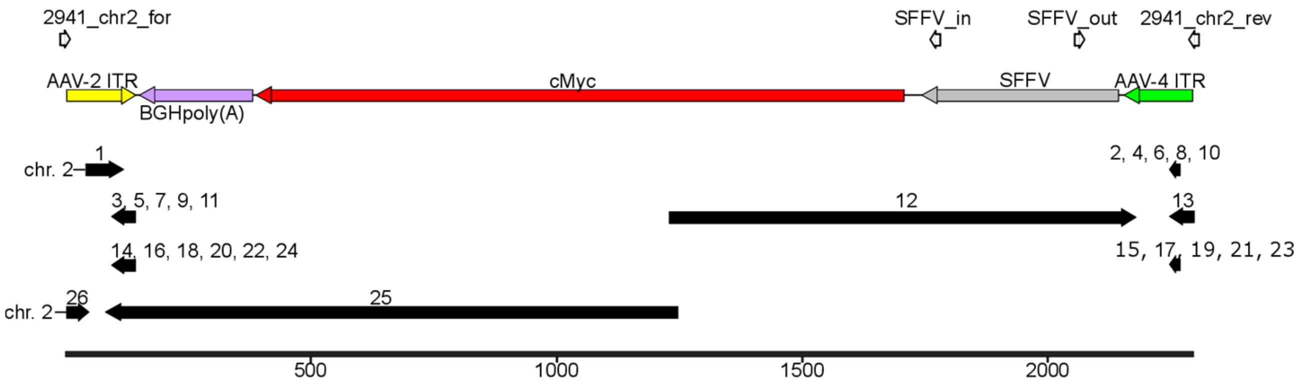

Supplementary Figure 10. Confirmation of an AAV IS in iPSC clone IVV-iPSC 14 (mouse #3, assay #2, Supplementary Table 5). (**a**) Image showing the reads surrounding the insertion site on chromosome 2 from target enrichment sequencing using the IGW browser. (**b**) Alignment of sequences from PCR products to the scAAV-SFFV-hCO-c-myc vector (colored arrows) using primers “2941_chr2_for” + “SFFV_in” and “2941_chr2_rev” + “SFFV_out” (white arrows). The resulting composition indicates vector rearrangements of 26 individual fragments (black arrows) including two loops of AAV-2 and AAV-4 ITR rearrangement (fragments 2-11 and 14-23). Numbers at the bottom indicate the size of the original vector in base pairs.

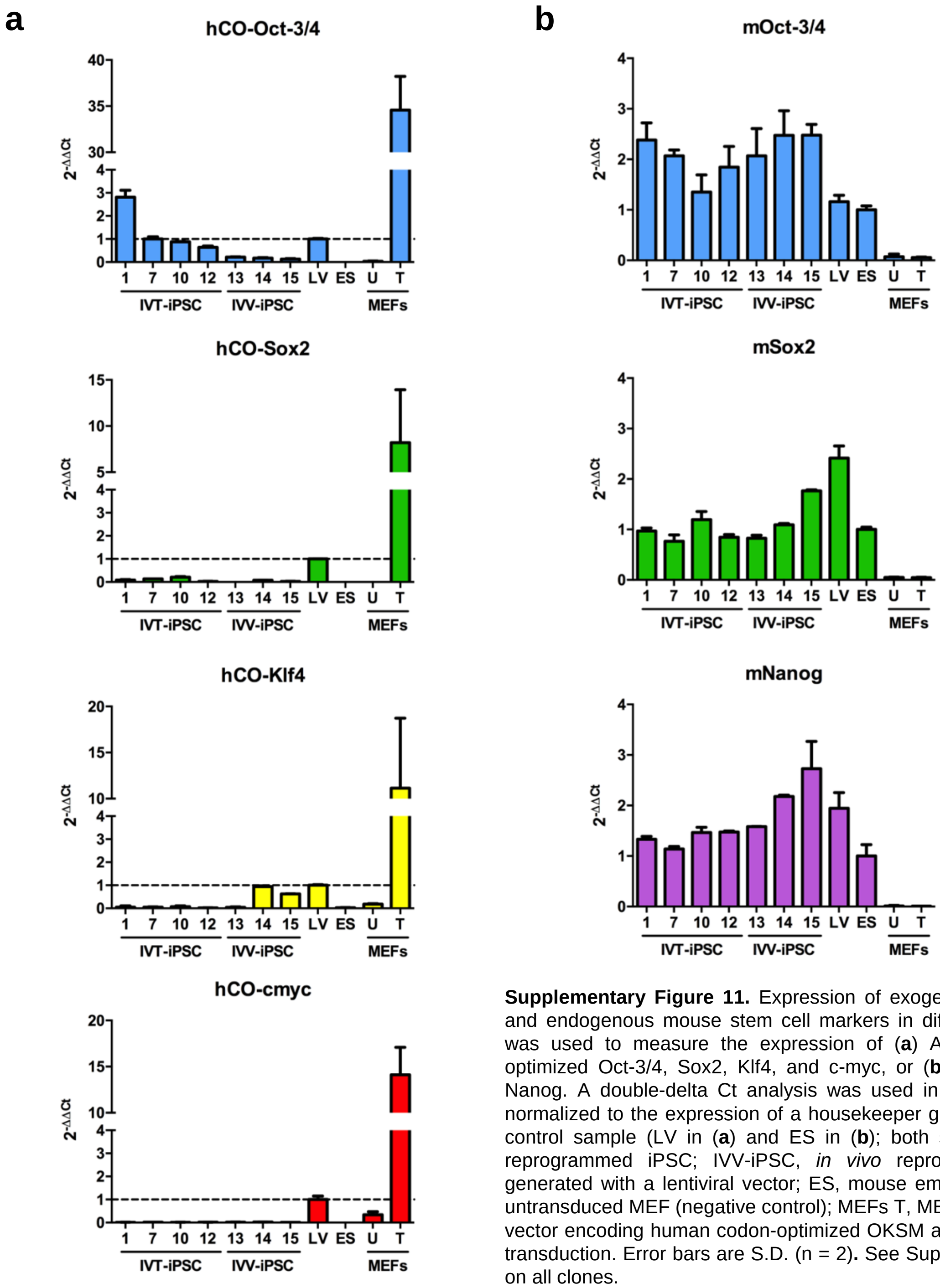

Supplementary Figure 11. Expression of exogenous reprogramming factors and endogenous mouse stem cell markers in different iPSC lines. qRT-PCR was used to measure the expression of (**a**) AAV-encoded human codon-optimized Oct-3/4, Sox2, Klf4, and c-myc, or (**b**) murine Oct-3/4, Sox2 and Nanog. A double-delta Ct analysis was used in which the values were first normalized to the expression of a housekeeper gene (Gapdh) and then to the control sample (LV in (**a**) and ES in (**b**); both set to 1). IVT-iPSC: *in vitro* reprogrammed iPSC; IVV-iPSC, *in vivo* reprogrammed iPSC; LV, iPSC generated with a lentiviral vector; ES, mouse embryonic stem cells; MEFs U, untransduced MEF (negative control); MEFs T, MEF transduced with a lentiviral vector encoding human codon-optimized OKSM and harvested four days post-transduction. Error bars are S.D. (n = 2)**.** See Supplementary Data 1 for details on all clones.

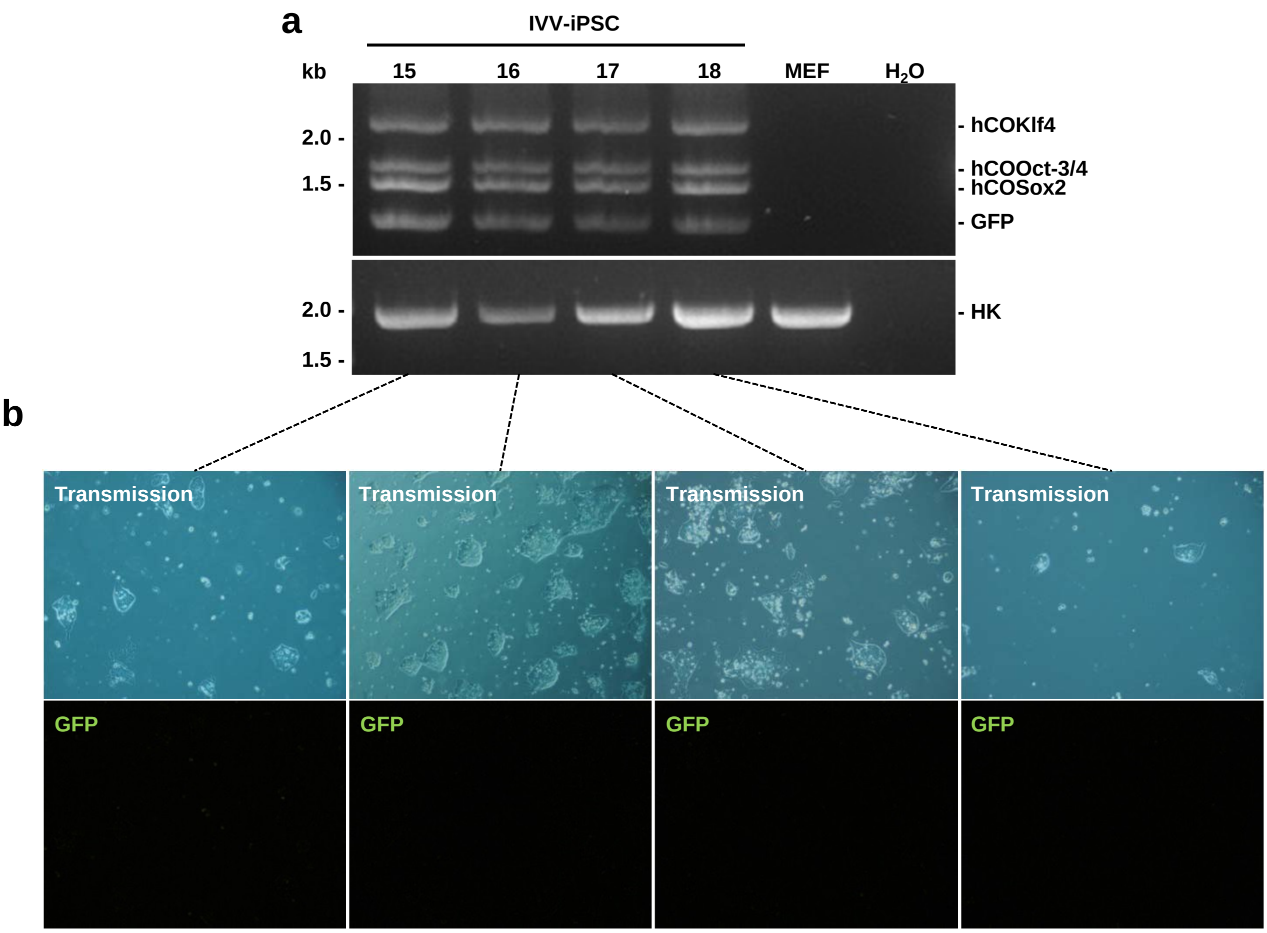

Supplementary Figure 12. Analysis of AAV vector traces in AAV-iPSC clones isolated from a teratoma of a mouse injected with a high dose of OKS (1×10^{12} vg) and a GFP vector substituting c-Myc. (**a**) PCR analysis of four independent AAV-iPSC clones isolated from the teratoma of mouse #1, assay #6 (Supplementary Table 5). Top gel: PCR with primers amplifying the whole cassette, from SFFV promoter to polyadenylation signal. Four distinct bands are detected, which correspond to the three AAV reprogramming vectors and the GFP vector used. Bottom gel: PCR to detect the *Gapdh* housekeeper (HK) gene. (**b**) Microscopy pictures showing no expression of GFP in the AAV-iPSC clones, despite the detection of AAV-SFFV-GFP vector persistence by PCR (see panel **a**). Images were taken at a 10x magnification with an Olympus PEN Lite E-PL3 camera attached with an Olympus Four Thirds adapter MMF-3 to an Olympus CKX41 microscope (Olympus, Hamburg, Germany).

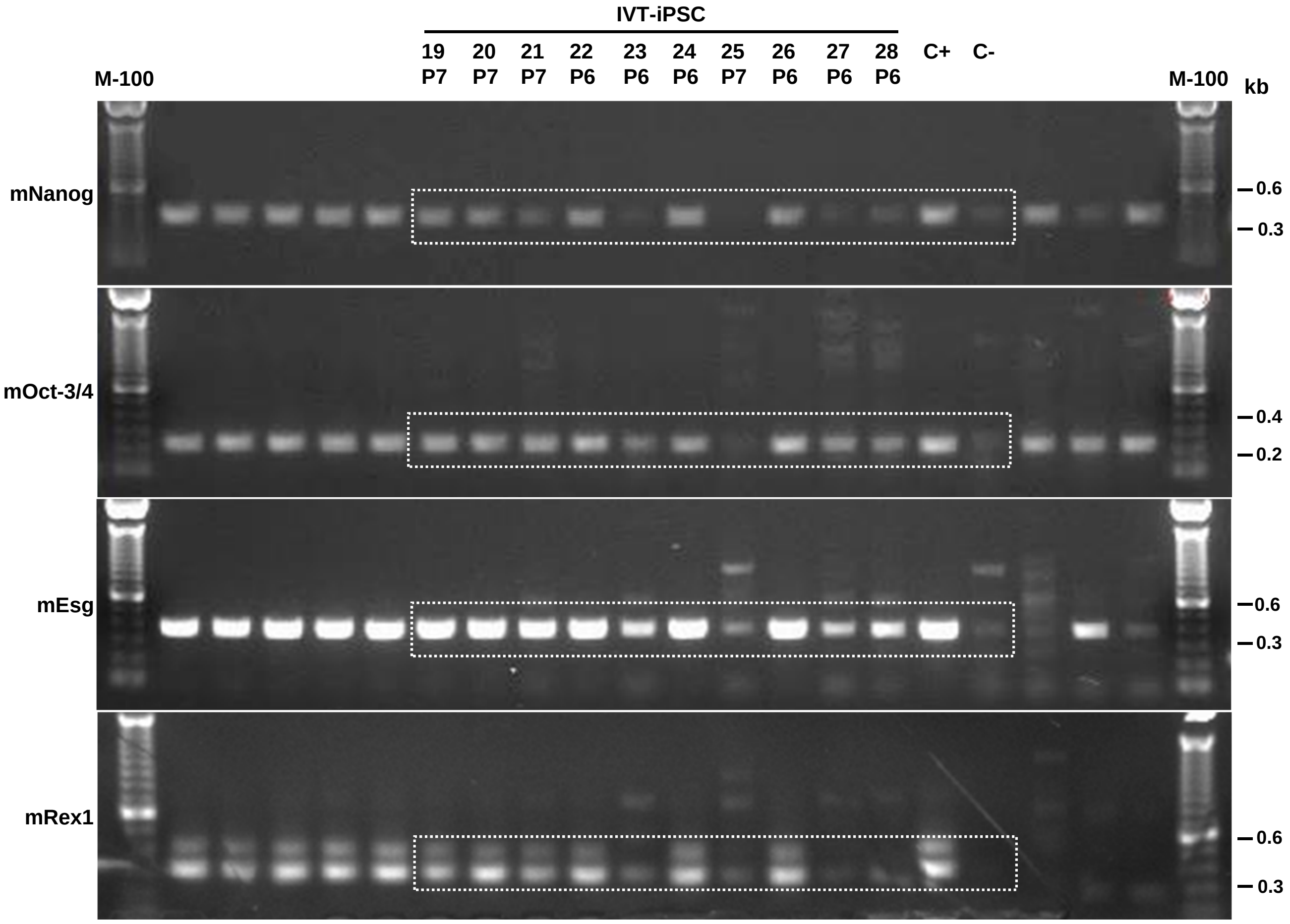

Figure 1c – first 4 gels (top to bottom)

Supplementary Figure 13. Uncropped images of the DNA gels shown in Figure 1c and 4a-b. The boxed regions were used in Figure 1c and 4a-b. DNA markers in the first and last lane of each gel are either a 100 bp (M-100) or a 1 kb (M-1) ladder (both Thermo Fisher Scientific). The positive controls (O, K, S, M) in the uncropped gel from Figure 4b are PCR products resulting from direct amplification of each of the four reprogramming factor cassettes from the original scAAV vector plasmids, obtained using primers SFFV_For_EcoRI and BGHpoly(A)_Rev (Supplementary Table 6).

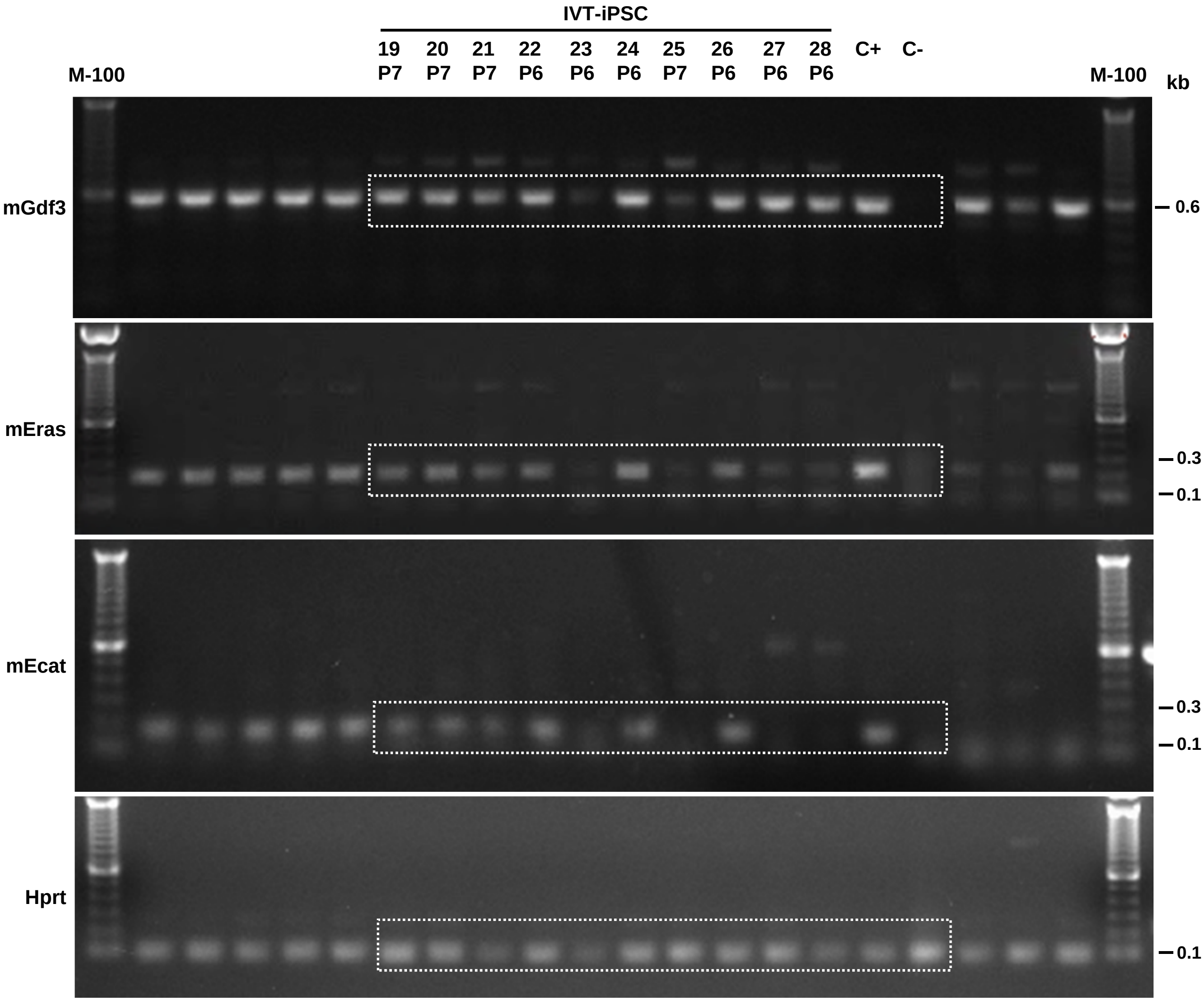

Figure 1c – second 4 gels (top to bottom)

Supplementary Figure 13 continued.

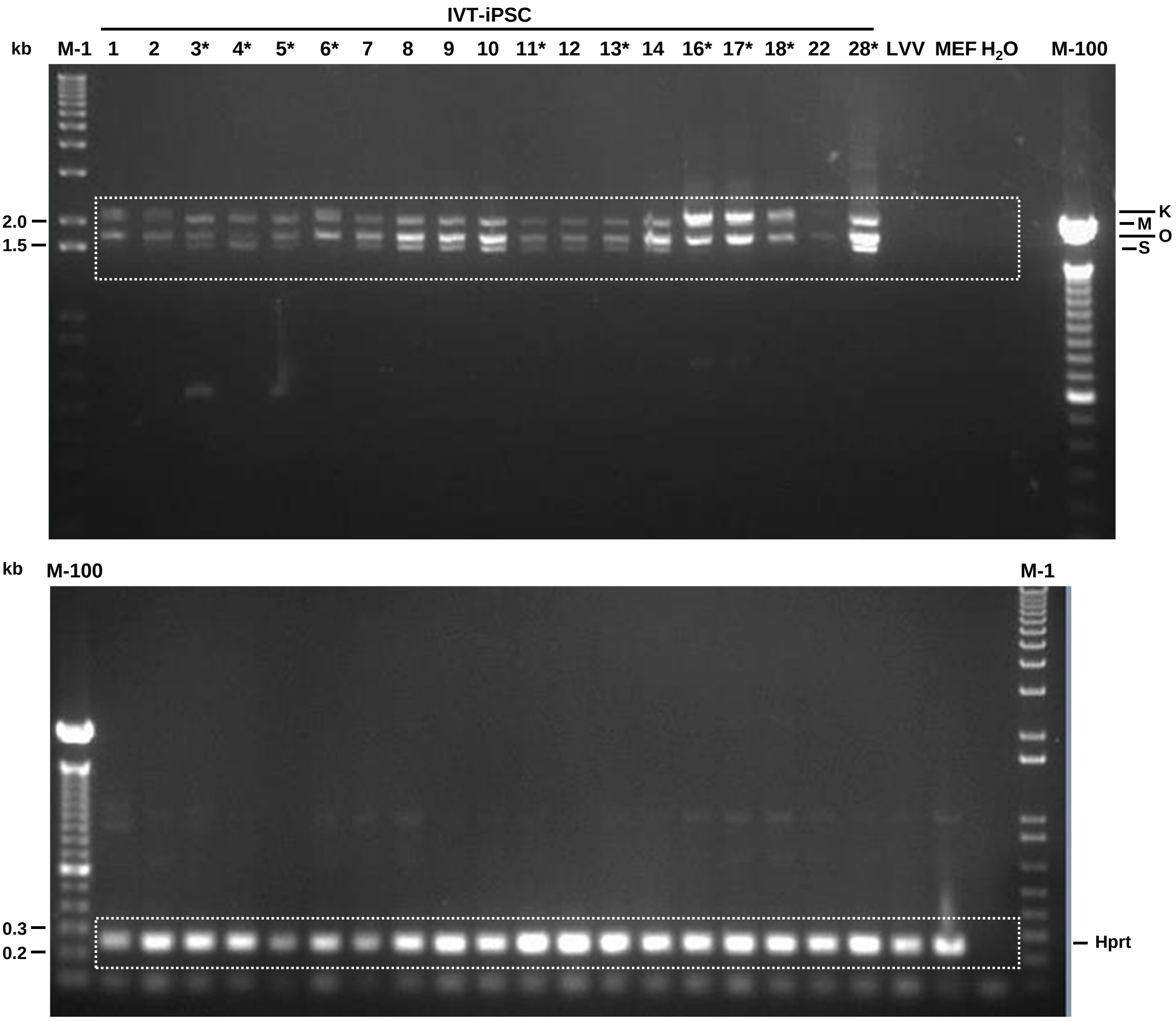

Figure 4a

Supplementary Figure 13 continued.

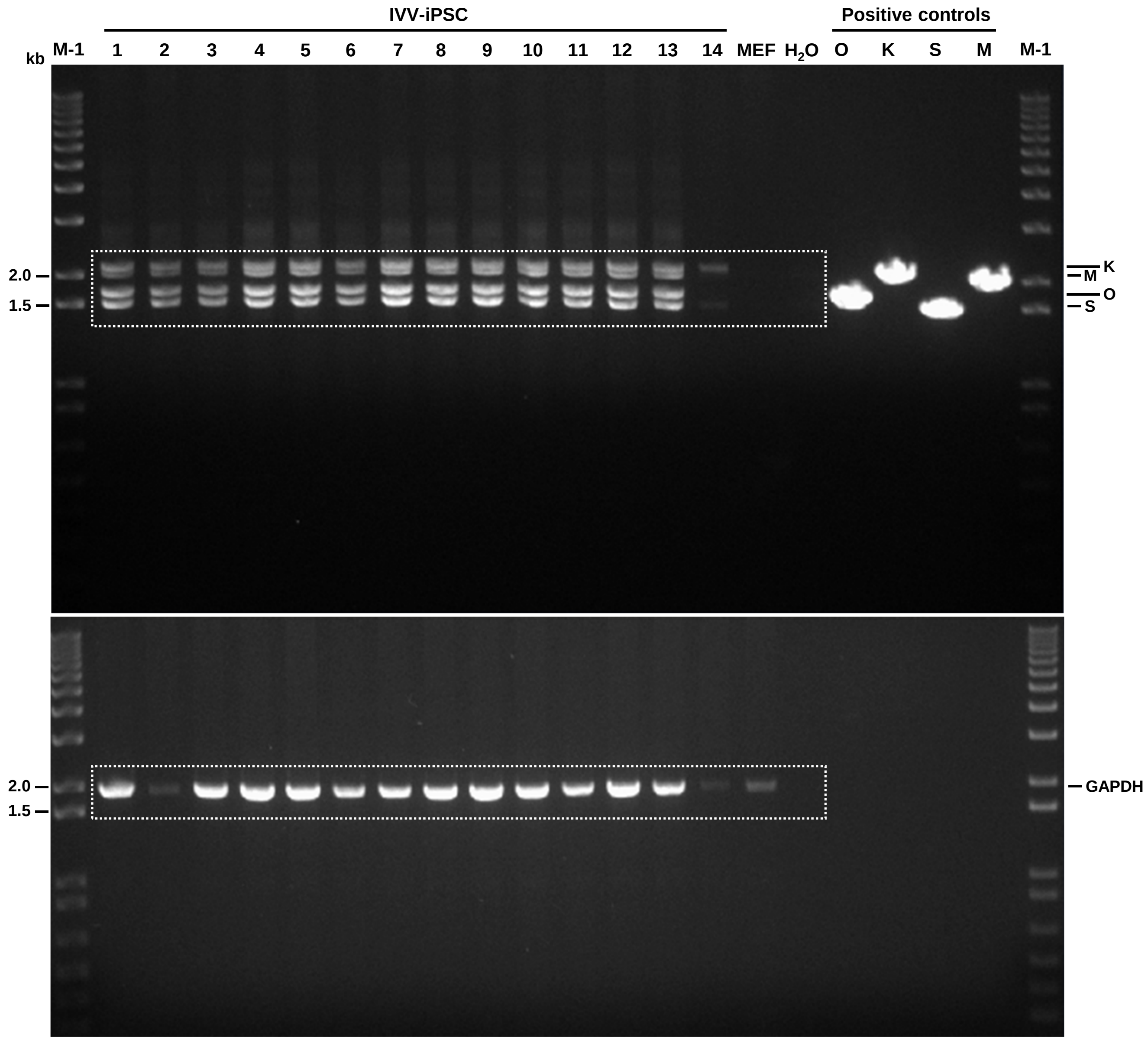

Figure 4b

Supplementary Figure 13 continued.

Supplementary Note 1. Risks and benefits of AAV vector integration in iPSC.

As noted in the main text, AAV vector DNA integration into mammalian host chromosomes is an intensely debated topic[1-6], and it is key to fully understand the mechanisms and consequences for AAV applications. Fortunately, thus far, the overwhelming majority of studies in small or large animals as well as in humans consistently suggests that long-term AAV vector persistence including genomic integration is safe and not associated with enhanced tumor formation[*e.g.*,7-15]. This was also concluded in a comprehensive *in vivo* study from the High lab that was specifically designed to favor the detection of possible tumorigenesis, by using over 130 mice, high AAV vector doses and long periods of follow-up, and that yielded no evidence for increased tumor generation due to AAV vector persistence and chromosomal integration[16]. This encouraging notion is further supported by our own current study where we also found no signs of AAV vector-induced formation of genuine tumors, not even at late time points (month 9 or 12) after injection of high doses of up to 1×10^{12} vg per vector and mouse.

Nonetheless, it is prudent and important to consider sporadic reports of hepatocellular carcinoma (HCC) in mice that may have been caused by AAV vector-mediated insertional mutagenesis[5, 6, 17-21]. Of particular interest in this regard is the *Rian* (*RNA imprinted and accumulated in the nucleus*) locus, a region on mouse chromosome 12 that is rich in small nucleolar (sno)RNAs and mi(cro)RNAs, including miR-341 whose dysregulation may be a key step in tumorigenesis[17, 19]. Importantly, this locus is only present in rodents, and integrations in the human homolog *DLK1-DIO3* (*delta-like homolog 1-deiodinase type* 3) were not observed in liver biopsies from AAV vector-treated patients[11], altogether alleviating concerns in humans. In our own study, too, integrations were neither detected in the *Rian* locus nor in any other genes (*e.g.*, *Hras1*, *Sos1*, *Fgf3*, *Fgf10* or *Raf1*) for which anecdotal reports suspected a link of AAV integration to genotoxicity or cancer formation in mice[17-20]. We did find integrations into exons of some other genes, however, for which the two mouse cancer databases that we queried noted an association with tumorigenesis (albeit not always unanimously, see column "Cancer-related"

in Supplementary Data 2). Notably, many of these genes play (at least) a dual role as they are concurrently involved in somatic cell reprogramming and stem cell renewal, such as *Esr1* (encoding the estrogen receptor ERα) and *Cnot3* (part of the Ccr4-Not deadenylase complex)[22, 23], or others (*Sox15*, *Sox21*, *Smad7*, *Ntn1*; Supplementary Data 2). Thus, and in view of the complete absence of tumors in all our animals, we believe that the putative role of these genes in cancer is neither related to, nor the cause of, their targeting by our vectors. Instead, we consider it much more likely that the dysregulation of these genes during AAV/OKSM-mediated cellular reprogramming altered their chromatin status and thereby facilitated AAV vector integration (see also below).

Finally important to note is that all prior studies that suspected a link between AAV integration and tumorigenesis found the risk of HCC development (if detected at all) to be (i) AAV vector dose-dependent, (ii) significantly higher for mice that were injected during the newborn period, and (iii) likewise elevated in mouse models of disease that were already prone to HCC formation, as compared to healthy, adult animals[17, 19-21]. This is further reassuring regarding the safety of AAV/OKSM vector applications, as therapeutic *in vivo* reprogramming seems most attractive for conditions that affect adults, such as degenerative or age-related ailments. In addition, future combinatorial improvements in vector efficiency, control and specificity that we outlined in the main text will help to lower vector doses, which will in turn minimize the risk of adverse AAV DNA integration in a clinical context. Despite this overall optimism, we clearly emphasize the necessity of additional studies which will characterize larger numbers of AAV vector integration sites in reprogrammed cells, and which will hopefully give a further statistical confirmation of the safety of the approach.

Apart from this qualitative analysis and comparison of AAV vector integration sites, we refrain from a thorough quantitative comparison of the present data with those reported before by others, for a number of reasons. Most notable are the facts that former results were obtained under largely varying conditions, which hampers or prevents a proper comparison to our new

data set. This includes the use and analysis of different species (cell lines versus mice, monkeys or humans), healthy versus diseased individuals, gender and age of animals or humans, organs (*e.g.*, liver or muscle), AAV serotypes, transgenes, regulatory elements, doses, or time points of analysis after vector administration, to name a few. Likewise, technologies for detection, quantification and characterization of AAV vector integration sites also vastly differed between the studies. Of note, the TES technology used here most likely exhibits superior sensitivity to most previously used methodologies. Finally, our study is additionally distinguished in that (i) we applied four different AAV vectors at the same time, whereas all prior studies that quantified integrations used only one; (ii) we investigated iPSC lines, while all others performed bulk analyses of cell pools or tissue samples; and (ii) we used scAAV, as opposed to the vast majority of previous work which studied ssAAV, including all reports on AAV integration sites in higher species (monkeys and humans).

Still, as pointed out in the main text, we note that there are similarities in the quantitative data on AAV integration in the present study and the literature, including results previously obtained by us (Schmidt lab) in samples from primates and humans treated with AAV vectors. Consistently, these data show between 3 and 225 unique integration sites per analyzed sample, including 29 to 225 in AAV1-treated human muscle[24], 4 to 196 in AAV5-treated primate and human tissues (comprising liver biopsies from clinical trials)[11], or 3 to 34 in AAV1- or AAV8-transduced primate muscle or liver[25], respectively. Similarly, others reported, for instance, 2.834 unique AAV integrations in 64 cancerous or healthy mouse livers, *i.e.*, an average of 44 integrations per sample. All these numbers are reminiscent of the 2 to 40 integration sites obtained in our iPSC clones (180 sites in 22 clones, *i.e.*, 8 on average), yet we re-emphasize that a direct comparison is very difficult due to the abovementioned manifold experimental differences between all studies and samples. Particularly crucial to realize is that the reported values were obtained with bulk samples (*e.g.*, tissue biopsies) and thus represent averages over large, heterogenous cell populations, whereas our data were gathered in iPSC lines that

presumably had a monoclonal origin. For this reason, it is also impossible to quantitatively compare the frequencies of AAV vector integration per overall cell number, because it was unknown in all previous studies how many cells per population were actually transduced, and because our current iPSC samples were most likely derived from single cells, as just noted.

Considering the function in the context of reprogramming, we believe that AAV integration is not causally related or required as all our vectors inserted at seemingly random positions and were eventually silenced (including the GFP control from Fig. 2f; Supplementary Fig. 12). Hence, rather than being an integral part of the reprogramming process, AAV vector insertion is probably a by-product of the chromatin relaxation and remodeling that occur during cellular dedifferentiation or rapid cell division. In line with this hypothesis, there are numerous reports that AAV DNA integrates into open, accessible chromatin with active genes and at pre-existing chromosome breaks instead of actively causing them[*e.g.*,16, 26-30]. In fact, the chromatin regions surrounding chromosome breaks are known to undergo extensive epigenetic remodeling[31, 32] which may in turn render them more susceptible to integration of exogenous (viral) DNA. Our conclusion is further consistent with the aforementioned finding that AAV vector integration frequencies, and thus also the risk of genotoxicity, are higher in livers of newborn mice that rapidly grow and are transcriptionally very active, as compared to mostly quiescent adult tissues. One specific example are data from the Venditti lab, who observed in their seminal 2015 study that genes such as albumin or α-fetoprotein, which are highly expressed in the neonatal liver, were most susceptible to AAV integration[5, 6, 19]. It is thus tempting to postulate that by stimulating chromatin remodeling and transcriptional activity, AAV/OKSM-induced cellular dedifferentiation mimics the situation in developing or cancerous tissues and thereby provides embryonic features which foster vector DNA integration.

Importantly, a serendipitous benefit of our *in vivo* reprogramming scheme is that it may serve as a novel and broadly useful model to study AAV integration biology including vector DNA integrity. In the future, it should be rewarding to exploit this new possibility to create

disseminated AAV vector insertions, by generating iPSC lines *in vitro* or *in vivo* for subsequent differentiation into various somatic cells, and by then studying the effects of the integrated ectopic DNA on cellular physiology and transformation. This will perfectly complement a battery of different *in vivo* models established by others before that likewise aimed at enhancing AAV DNA integration frequencies, such as vector injection into neonatal mice[17], clonal selection of stably transduced hepatocytes in the HTI (hereditary tyrosinemia I) mouse[33], murine hematopoietic stem cell transduction and serial bone marrow transplants[34], or high-dose liver-directed AAV gene transfer and long-term follow-up analyses in adult mice[16]. We also note complementing experimental strategies to study the sequelae of AAV vector integrations into tumor-associated genes, such as the intentional introduction of a strong enhancer/promoter into the mouse *Rian* locus via AAV gene targeting[35]. As compared to all these existing models of AAV vector integration and its consequences, a particular benefit of our new iPSC-based approach is that it is much more versatile, because it can be applied in virtually any cell that is amenable to AAV transduction, and because the resulting iPSC can be differentiated into manifold cell types. Moreover, as opposed to the majority of previous work that was performed in heterologous cell populations such as tumor samples or tissue biopsies, the iPSC lines generated with the vectors reported here are expected to be far more homogenous, due to their presumed monoclonal origin. This will allow to perform large-scale surveys under stringent conditions and in various cell types, which will expand our knowledge of the factors that govern AAV vector integration, such as sequence motifs or chromatin marks, as well as of mammalian chromosome biology in general.

In this context, it will be particularly interesting to further harness the new TES technology that we used in this study as a powerful alternative to the established nrLAM-PCR approach. This is because TES additionally allows for the detection and analysis of internal AAV vector breakage and rearrangement events, as exemplified here in Supplementary Figure 10. Our finding of substantial vector rearrangement (comprising 26 individual fragments in the shown

example) is in line with our observations that we have recently made using multiplexed LAM-PCR in NHP and human samples[11]. Taken together, our recent and new data strongly imply that AAV vector integration mechanisms are far more complex than previously anticipated, as they can involve recombination events that occur between distant vector regions and that do not necessarily comprise the AAV ITR regions. The feasibility to create large numbers of AAV DNA integrations using our novel AAV/OKSM vectors should prove very valuable for future attempts to study the nature and biogenesis of these heavily rearranged vector genomes, including a possible role of internal homologies for recombination and chromosomal insertion. This will not only improve our fundamental understanding of AAV biology, but it will eventually also help to inform academic and industrial researchers contemplating the use of AAV vectors for human gene therapy.

1. Berns, K.I. *et al*. Adeno-Associated Virus Type 2 and Hepatocellular Carcinoma? *Hum. Gene Ther.* **26**, 779-781 (2015).
2. Buning, H. & Schmidt, M. Adeno-associated Vector Toxicity-To Be or Not to Be? *Mol. Ther.* **23**, 1673-1675 (2015).
3. Nault, J.C. *et al*. Recurrent AAV2-related insertional mutagenesis in human hepatocellular carcinomas. *Nat. Genet.* **47**, 1187-1193 (2015).
4. Logan, G.J. *et al*. Identification of liver-specific enhancer-promoter activity in the 3' untranslated region of the wild-type AAV2 genome. *Nat. Genet.* **49**, 1267-1273 (2017).
5. Chandler, R.J., LaFave, M.C., Varshney, G.K., Burgess, S.M. & Venditti, C.P. Genotoxicity in Mice Following AAV Gene Delivery: A Safety Concern for Human Gene Therapy? *Mol. Ther.* **24**, 198-201 (2016).
6. Chandler, R.J., Sands, M.S. & Venditti, C.P. Recombinant Adeno-Associated Viral Integration and Genotoxicity: Insights from Animal Models. *Hum. Gene Ther.* **28**, 314-322 (2017).

7. Nichols, T.C. *et al*. Protein replacement therapy and gene transfer in canine models of hemophilia A, hemophilia B, von willebrand disease, and factor VII deficiency. *ILAR J.* **50**, 144-167 (2009).

8. Niemeyer, G.P. *et al*. Long-term correction of inhibitor-prone hemophilia B dogs treated with liver-directed AAV2-mediated factor IX gene therapy. *Blood* **113**, 797-806 (2009).

9. Bell, P. *et al*. Analysis of tumors arising in male B6C3F1 mice with and without AAV vector delivery to liver. *Mol. Ther.* **14**, 34-44 (2006).

10. Nathwani, A.C. *et al*. Long-term safety and efficacy of factor IX gene therapy in hemophilia B. *N. Engl. J. Med.* **371**, 1994-2004 (2014).

11. Gil-Farina, I. *et al*. Recombinant AAV Integration Is Not Associated With Hepatic Genotoxicity in Nonhuman Primates and Patients. *Mol. Ther.* **24**, 1100-1105 (2016).

12. Paneda, A. *et al*. Safety and liver transduction efficacy of rAAV5-cohPBGD in nonhuman primates: a potential therapy for acute intermittent porphyria. *Hum. Gene Ther.* **24**, 1007-1017 (2013).

13. Li, S. *et al*. Efficient and Targeted Transduction of Nonhuman Primate Liver With Systemically Delivered Optimized AAV3B Vectors. *Mol. Ther.* **23**, 1867-1876 (2015).

14. D'Avola, D. *et al*. Phase I open label liver-directed gene therapy clinical trial for acute intermittent porphyria. *J. Hepatol.* **65**, 776-783 (2016).

15. Nathwani, A.C. *et al*. Long-term safety and efficacy following systemic administration of a self-complementary AAV vector encoding human FIX pseudotyped with serotype 5 and 8 capsid proteins. *Mol. Ther.* **19**, 876-885 (2011).

16. Li, H. *et al*. Assessing the potential for AAV vector genotoxicity in a murine model. *Blood* **117**, 3311-3319 (2011).

17. Donsante, A. *et al*. AAV vector integration sites in mouse hepatocellular carcinoma. *Science* **317**, 477 (2007).

18. Donsante, A. *et al*. Observed incidence of tumorigenesis in long-term rodent studies of rAAV vectors. *Gene Ther.* **8**, 1343-1346 (2001).

19. Chandler, R.J. *et al*. Vector design influences hepatic genotoxicity after adeno-associated virus gene therapy. *J. Clin. Invest.* **125**, 870-880 (2015).

20. Rosas, L.E. *et al*. Patterns of scAAV vector insertion associated with oncogenic events in a mouse model for genotoxicity. *Mol. Ther.* **20**, 2098-2110 (2012).

21. Zhong, L. *et al*. Recombinant adeno-associated virus integration sites in murine liver after ornithine transcarbamylase gene correction. *Hum. Gene Ther.* **24**, 520-525 (2013).

22. Nakada, D. *et al*. Oestrogen increases haematopoietic stem-cell self-renewal in females and during pregnancy. *Nature* **505**, 555-558 (2014).

23. Zheng, X. *et al*. CNOT3-Dependent mRNA Deadenylation Safeguards the Pluripotent State. *Stem Cell Reports* **7**, 897-910 (2016).

24. Kaeppel, C. *et al*. A largely random AAV integration profile after LPLD gene therapy. *Nat. Med.* **19**, 889-891 (2013).

25. Nowrouzi, A. *et al*. Integration frequency and intermolecular recombination of rAAV vectors in non-human primate skeletal muscle and liver. *Mol. Ther.* **20**, 1177-1186 (2012).

26. Miller, D.G., Petek, L.M. & Russell, D.W. Adeno-associated virus vectors integrate at chromosome breakage sites. *Nat. Genet.* **36**, 767-773 (2004).

27. Miller, D.G. *et al*. Large-scale analysis of adeno-associated virus vector integration sites in normal human cells. *J. Virol.* **79**, 11434-11442 (2005).

28. Nakai, H. *et al*. AAV serotype 2 vectors preferentially integrate into active genes in mice. *Nat. Genet.* **34**, 297-302 (2003).

29. Huser, D., Gogol-Doring, A., Chen, W. & Heilbronn, R. Adeno-associated virus type 2 wild-type and vector-mediated genomic integration profiles of human diploid fibroblasts analyzed by third-generation PacBio DNA sequencing. *J. Virol.* **88**, 11253-11263 (2014).

30. Huser, D. *et al*. Integration preferences of wildtype AAV-2 for consensus rep-binding sites at numerous loci in the human genome. *PLoS Pathog.* **6**, e1000985 (2010).

31. Cann, K.L. & Dellaire, G. Heterochromatin and the DNA damage response: the need to relax. *Biochem. Cell Biol.* **89**, 45-60 (2011).

32. Jeggo, P.A. & Downs, J.A. Roles of chromatin remodellers in DNA double strand break repair. *Exp. Cell Res.* **329**, 69-77 (2014).

33. Nakai, H. *et al*. Large-scale molecular characterization of adeno-associated virus vector integration in mouse liver. *J. Virol.* **79**, 3606-3614 (2005).

34. Han, Z. *et al*. Stable integration of recombinant adeno-associated virus vector genomes after transduction of murine hematopoietic stem cells. *Hum. Gene Ther.* **19**, 267-278 (2008).

35. Wang, P.R. *et al*. Induction of hepatocellular carcinoma by in vivo gene targeting. *Proc. Natl. Acad. Sci. USA* **109**, 11264-11269 (2012).
