# Supplementary material for: AAV vector-mediated in vivo reprogramming into pluripotency
Source: Nat Commun. 2018 Jul 9;9:2651. doi: 10.1038/s41467-018-05059-x (PMC6037684; doi:10.1038/s41467-018-05059-x)
Supplement: Supplementary file 2 — Description of Additional Supplementary Files [file 41467_2018_5059_MOESM2_ESM.pdf]

Description of Additional Supplementary Files

File Name: Supplementary Data 1
Description: An overview of all iPSC clones that were generated in this study in vitro (IVT, tab "In vitro") or in vivo (IVV, tab "In vivo").

File Name: Supplementary Data 2
Description: A comprehensive characterization of AAV vector integration sites in partially or fully reprogrammed iPSC clones generated in this work.

File Name: Supplementary Movie 1
Description: MEF-derived iPSC colonies that spontaneously differentiated into beating cells (most likely cardiomyocytes) in the absence of LIF. The MEF were reprogrammed into iPSC using AAV-DJ SFFV-hCO-O/K/S/M vectors at a MOI of 1×104 and a 4:1:1:1 O:K:S:M stoichiometry. The beating area was recorded with an AmScope microscope digital camera MD800E (AmScope, Irvine, CA, USA) attached to an Olympus CKX41 microscope (Olympus, Hamburg, Germany).

File Name: Supplementary Movie 2
Description: A second, independent example of MEF-derived iPSC colonies that differentiated into beating cells when maintained in the absence of LIF. For details on the reprogramming process and movie acquisition, please see Supplementary Movie 1.
